# Supplementary material for: Temporal and regional trends of antibiotic use in long-term aged care facilities across 39 countries, 1985-2019: Systematic review and meta-analysis
Source: PLoS One. 2021 Aug 23;16(8):e0256501. doi: 10.1371/journal.pone.0256501 (PMC8382177; doi:10.1371/journal.pone.0256501)
Supplement: S3 File — (DOCX) [file pone.0256501.s003.docx]

**S3 File: Search strategy**

**MEDLINE Search strategy (via OvidSP)**

Date searched: April 2021

| **Search ID#** | **Search Terms** |
| --- | --- |
| 1 | exp residential facilities/ |
| 2 | exp long-term care/ |
| 3 | residential facilit*.ti,ab. |
| 4 | aged care.ti,ab. |
| 5 | assisted living facilit*.ti,ab. |
| 6 | homes for the aged.ti,ab. |
| 7 | nursing home*.ti,ab. |
| 8 | skilled nursing facilit*.ti,ab. |
| 9 | long-term care.ti,ab. |
| 10 | longterm care.ti,ab. |
| 11 | exp *anti-bacterial agents/tu or exp *anti-infective agents, urinary/tu |
| 12 | exp *antimicrobial stewardship/ |
| 13 | exp aminoglycosides/tu |
| 14 | exp fluoroquinolones/tu |
| 15 | exp lactams/tu |
| 16 | exp methenamine/tu |
| 17 | exp nitrofurantoin/tu |
| 18 | exp sulfonamides/tu |
| 19 | exp tetracyclines/tu |
| 20 | exp trimethoprim/tu |
| 21 | exp vancomycin/tu |
| 22 | antibiot*.ti,ab. |
| 23 | anti-biot*.ti,ab. |
| 24 | anti-microb*.ti,ab. |
| 25 | antimicrob*.ti,ab. |
| 26 | anti-infective*.ti,ab. |
| 27 | antiinfective*.ti,ab. |
| 28 | anti-bacterial*.ti,ab. |
| 29 | antibacterial*.ti,ab. |
| 30 | (amoxcillin or amoxicillin or amoxycillin).ti,ab. |
| 31 | aminoglycoside*.ti,ab. |
| 32 | ampicillin.ti,ab. or ampycillin.ti,ab. |
| 33 | beta-lactam*.ti,ab. |
| 34 | betalactam*.ti,ab. |
| 35 | carbapenem*.ti,ab. |
| 36 | cefalexin.ti,ab. or cephalexin.ti,ab. |
| 37 | cefalosporin*.ti,ab. or cephalosporin*.ti,ab. |
| 38 | ciprofloxacin.ti,ab. |
| 39 | clavulan*.ti,ab. |
| 40 | co-amoxiclav.ti,ab. |
| 41 | cotrimoxazole.ti,ab. |
| 42 | doxycycline.ti,ab. |
| 43 | flucloxacillin.ti,ab. |
| 44 | fluoroquinolone*.ti,ab. |
| 45 | levofloxacin.ti,ab. |
| 46 | metheneamine.ti,ab. |
| 47 | nitrofurantoin.ti,ab. |
| 48 | pivmecillinam.ti,ab. |
| 49 | quinolone*.ti,ab. |
| 50 | trimethoprim*.ti,ab. |
| 51 | vancomycin.ti,ab. |
| 52 | or/1-10 |
| 53 | or/11-51 |
| 54 | 52 and 53 |
| 55 | limit 54 to yr="1990 -current" |
| 56 | limit 55 to english language |
| 57 | limit 56 to humans |
| 58 | limit 57 to journal article |

**EMBASE Search Strategy (via OvidSP)**

Date searched: April 2021

| **Search ID#** | **Search Terms** |
| --- | --- |
| 1 | exp residential home/ |
| 2 | exp assisted living facilities/ |
| 3 | exp homes for the aged/ |
| 4 | exp nursing homes/ |
| 5 | residential facilit*.ti,ab. |
| 6 | aged care.ti,ab. |
| 7 | assisted living facilit*.ti,ab. |
| 8 | homes for the aged.ti,ab. |
| 9 | nursing home*.ti,ab. |
| 10 | skilled nursing facilit*.ti,ab. |
| 11 | long-term care.ti,ab. |
| 12 | longterm care.ti,ab. |
| 13 | exp *anti-infective agents/ |
| 14 | exp *antimicrobial stewardship/ |
| 15 | exp *antimicrobial therapy/ |
| 16 | exp *antibiotic agent/ |
| 17 | antibiot*.ti,ab. |
| 18 | anti-biot*.ti,ab. |
| 19 | anti-microb*.ti,ab. |
| 20 | antimicrob*.ti,ab. |
| 21 | anti-infective*.ti,ab. |
| 22 | antiinfective*.ti,ab. |
| 23 | anti-bacterial*.ti,ab. |
| 24 | antibacterial*.ti,ab. |
| 25 | exp aminoglycosides/dt |
| 26 | exp fluoroquinolones/dt |
| 27 | exp lactams/dt |
| 28 | exp methenamine/dt |
| 29 | exp nitrofurantoin/dt |
| 30 | exp sulfonamides/dt |
| 31 | exp tetracyclines/dt |
| 32 | exp trimethoprim/dt |
| 33 | exp vancomycin/dt |
| 34 | aminoglycoside*.ti,ab. |
| 35 | carbapenem*.ti,ab. |
| 36 | cefalexin.ti,ab. or cephalexin.ti,ab. |
| 37 | cefalosporin*.ti,ab. or cephalosporin*.ti,ab. |
| 38 | ciprofloxacin.ti,ab. |
| 39 | clavulan*.ti,ab. |
| 40 | co-amoxiclav.ti,ab. |
| 41 | cotrimoxazole.ti,ab. |
| 42 | fluoroquinolone*.ti,ab. |
| 43 | levofloxacin.ti,ab. |
| 44 | quinolone*.ti,ab. |
| 45 | vancomycin.ti,ab. |
| 46 | or/1-12 |
| 47 | or/13-45 |
| 48 | 46 and 47 |
| 49 | limit 48 to english language |
| 50 | limit 49 to yr="1990 -current" |
| 51 | limit 50 to human |
| 52 | limit 51 to (conference abstract or "conference review" or editorial or erratum or letter or note or "review") |
| 53 | 51 not 52 |

**Medline (via PubMed)**

Date searched: April 2021

Search strategy: As for Medline.
