# Supplementary material for: Temporal and regional trends of antibiotic use in long-term aged care facilities across 39 countries, 1985-2019: Systematic review and meta-analysis
Source: PLoS One. 2021 Aug 23;16(8):e0256501. doi: 10.1371/journal.pone.0256501 (PMC8382177; doi:10.1371/journal.pone.0256501)
Supplement: S4 File — (DOCX) [file pone.0256501.s004.docx]

**S4 File: Quality assessment score for studies as assessed against Joanna Briggs Institute Critical Appraisal Tool for Prevalence Studies**

| **Author** | **Criteria 1** | **Criteria 2** | **Criteria 3** | **Criteria 4** | **Criteria 5** | **Criteria 6** | **Criteria 7** | **Criteria 8** | **Criteria 9** | **Score** |
| --- | --- | --- | --- | --- | --- | --- | --- | --- | --- | --- |
| Alberg, 2017[1] | Y | Y | Y | N | N | Y | U | N | N | Poor |
| acNAPS, 2016[2] | Y | Y | Y | Y | Y | Y | Y | N | Y | Good |
| acNAPS, 2017[3, 4] | Y | Y | Y | Y | Y | Y | Y | N | Y | Good |
| acNAPS, 2018[5] | Y | Y | Y | Y | Y | Y | Y | N | Y | Good |
| acNAPS, 2019[6] | Y | Y | Y | Y | Y | Y | Y | N | Y | Good |
| acNAPS, 2020[7] | Y | Y | Y | N | Y | Y | Y | N | Y | Good |
| Barney, 2019[8] | Y | Y | N | N | Y | Y | Y | Y | Y | Good |
| Benoit, 2008[9] | Y | Y | Y | Y | Y | Y | U | N | Y | Good |
| Blix, 2007[10] | Y | Y | Y | N | Y | Y | Y | Y | Y | Good |
| Blix, 2010[11] | Y | Y | Y | N | Y | Y | Y | N | Y | Good |
| Boivin, 2013[12] | Y | Y | Y | N | Y | Y | Y | Y | Y | Good |
| Cowan, 2016[13] | N | Y | U | N | Y | Y | U | N | Y | Poor |
| Daneman, 2011[14] | Y | Y | Y | Y | Y | Y | Y | Y | Y | Good |
| Daneman, 2013[15] | Y | Y | Y | Y | Y | Y | Y | Y | Y | Good |
| Daneman, 2015[16] | Y | Y | Y | Y | Y | Y | Y | Y | Y | Good |
| Daneman, 2017[17] | Y | Y | Y | Y | Y | Y | Y | Y | Y | Good |
| Drinka, 2004[18] | N | Y | N | Y | Y | Y | Y | N | Y | Fair |
| Eikelenboom-Boskamp, 2019[19] | Y | Y | Y | N | Y | Y | Y | Y | Y | Good |
| ESAC -1[20-26] | Y | Y | Y | N | Y | Y | Y | Y | Y | Good |
| ESAC-2[23, 24] | Y | Y | Y | N | Y | Y | Y | Y | Y | Good |
| ESAC-3[24] | Y | Y | Y | N | Y | Y | Y | Y | Y | Good |
| Fagan, 2012[27] | Y | Y | Y | N | Y | Y | Y | N | Y | Good |
| Felsen, 2020[28] | N | Y | N | N | N | Y | Y | N | Y | Poor |
| Fleet, 2014[29] | Y | U | Y | N | Y | Y | Y | N | Y | Fair |
| Gillespie, 2015[30] | Y | Y | Y | Y | N | Y | Y | Y | N | Good |
| HALT-1[31-39] | Y | Y | Y | N | Y | Y | Y | Y | Y | Good |
| HALT-2[35-37] | Y | Y | Y | N | Y | Y | Y | Y | Y | Good |
| HALT-3[32-34] | Y | Y | Y | N | Y | Y | Y | Y | Y | Good |
| Heudorf, 2012[40] | Y | Y | Y | Y | Y | Y | Y | N | Y | Good |
| Ishikane, 2020[41] | N | Y | N | N | Y | Y | Y | N | Y | Fair |
| Jump, 2012[42] | N | Y | N | N | Y | Y | Y | Y | Y | Fair |
| Katz, 1990[43] | N | Y | N | Y | Y | Y | Y | N | Y | Fair |
| Kabbani, 2019[44] | Y | Y | Y | N | Y | Y | Y | Y | Y | Good |
| Lee, 1992[45] | Y | Y | N | N | Y | Y | Y | N | Y | Fair |
| Lee, 1996[46] | N | Y | N | Y | Y | N | Y | N | Y | Fair |
| Loeb, 2005[47] | Y | Y | Y | N | Y | U | Y | N | Y | Fair |
| Marquet, 2015[48] | Y | U | Y | N | Y | Y | Y | N | U | Fair |
| Marra, 2017[49] | Y | Y | Y | Y | Y | Y | Y | N | Y | Good |
| Mayne, 2018[50] | N | Y | N | Y | Y | Y | Y | N | Y | Fair |
| Monette, 2007[51] | N | Y | N | N | Y | Y | Y | N | U | Poor |
| Montgomery, 1995[52] | Y | Y | Y | N | Y | Y | Y | N | Y | Good |
| Moro, 2007[53] | Y | Y | Y | Y | U | Y | Y | Y | U | Good |
| Myolette, 1999[54] | N | Y | N | N | Y | Y | U | Y | Y | Fair |
| Mylotte, 2005[55] | N | Y | N | N | Y | Y | U | Y | Y | Fair |
| Natsch, 1998[56] | N | Y | N | N | Y | Y | U | N | Y | Fair |
| Olsho, 2013[57] | Y | Y | Y | Y | Y | U | Y | N | Y | Good |
| Pakyz, 2010[58] | Y | Y | Y | N | Y | Y | Y | Y | Y | Good |
| Pluss-Suard, 2019[59] | Y | Y | Y | N | Y | N | U | N | Y | Fair |
| Raban, 2020[60] | Y | Y | Y | Y | Y | Y | Y | Y | Y | Good |
| Rahme, 2016[61] | N | Y | N | N | Y | Y | Y | Y | Y | Fair |
| Roukens, 2017[62] | Y | Y | Y | N | Y | Y | U | N | U | Fair |
| Rummukainen, 2009[63] | Y | Y | Y | Y | Y | N | U | N | Y | Fair |
| Rummukainen, 2013[64] | Y | Y | Y | Y | Y | Y | U | Y | Y | Good |
| Saxena, 2019[65] | Y | Y | Y | N | Y | Y | Y | N | Y | Good |
| Selcuk, 2018[66] | Y | Y | N | N | Y | N | Y | N | Y | Fair |
| Selcuk, 2019[67] | N | Y | U | N | Y | Y | Y | N | Y | Fair |
| Sloane, 2014[68] | N | Y | N | N | Y | Y | Y | N | Y | Fair |
| Sloane, 2019[69] | Y | Y | Y | Y | Y | U | U | N | Y | Fair |
| Sloane, 2020[69] | Y | U | Y | Y | Y | Y | U | N | U | Fair |
| Sluggett, 2020[70] | Y | Y | Y | Y | Y | Y | Y | Y | Y | Good |
| Smith, 2013[71] | Y | Y | Y | N | Y | Y | Y | Y | Y | Good |
| Smith, 2020[72] | Y | Y | Y | Y | N | Y | Y | Y | Y | Good |
| Song, 2021[73] | Y | Y | Y | Y | Y | Y | Y | Y | Y | Good |
| Stepan, 2018[74] | Y | Y | Y | N | Y | Y | U | Y | Y | Good |
| Stuart, 2012[75] | N | Y | N | Y | Y | Y | U | N | Y | Fair |
| Stuart, 2015[76] | N | Y | N | N | Y | Y | Y | N | U | Poor |
| Sundvall, 2015[77] | Y | U | Y | Y | Y | Y | Y | N | Y | Good |
| Takito, 2020[78] | N | Y | N | N | U | U | U | N | U | Poor |
| Taxis, 2017[79] | Y | Y | Y | N | Y | Y | Y | N | Y | Good |
| Temime, 2018[80] | Y | U | Y | N | U | Y | U | N | Y | Poor |
| Thompson, 2016[81] | N | Y | N | N | N | Y | Y | Y | U | Poor |
| Thompson, 2021[82] | Y | Y | Y | Y | Y | Y | Y | Y | N | Good |
| Thornley, 2019a[83] | Y | Y | Y | N | Y | Y | Y | N | Y | Good |
| Thornley, 2019b[84] | Y | Y | Y | N | Y | Y | Y | N | Y | Good |
| van Buul, 2015[85] | Y | Y | N | Y | Y | Y | Y | N | Y | Good |
| Warren, 1991[86] | Y | U | Y | N | Y | Y | Y | N | U | Fair |
| Wu, 2015[87] | N | Y | U | Y | Y | Y | Y | Y | Y | Good |
| Zimmerman, 2014[88] | N | Y | N | Y | Y | Y | Y | N | Y | Fair |

Y is ‘yes’, i.e. criterion met. N is ‘no’, i.e. criterion not met. U is ‘unclear’, i.e. unclear whether criterion met.

**References**

1. Alberg T, Holen O, Blix HS, Lindbaek M, Bentele H, Eriksen HM. Antibiotic use and infections in nursing homes. Tidsskrift for Den Norske Laegeforening. 2017;137(5):357-61.

2. National Centre for Antimicrobial Stewardship, Australian Commission on Safety and Quality in Health Care. Antimicrobial prescribing and infections in Australian residential aged care facilities: results of the 2015 Aged Care National Antimicrobial Prescribing Survey pilot Sydney: ACSQHC; 2016 [Available from: <https://www.safetyandquality.gov.au/sites/default/files/migrated/Antimicrobial-prescribing-and-infections-in-Australian-residential-aged-care-facilities-2015-acNAPS.pdf>.

3. National Centre for Antimicrobial Stewardship, Australian Commission on Safety and Quality in Health Care. Aged Care National Antimicrobial Prescribing Survey 2016. Sydney: ACSQHC; 2017.

4. Bennett N, Imam N, James R, Chen C, Bull A, Thursky K, et al. Prevalence of infections and antimicrobial prescribing in Australian aged care facilities: Evaluation of modifiable and nonmodifiable determinants. American Journal of Infection Control. 2018;46(10):1148-53.

5. National Centre for Antimicrobial Stewardship, Australian Commission on Safety and Quality in Health Care. Antimicrobial Prescribing and Infections in Australian Aged Care Homes: Results of the 2017 Aged Care National Antimicrobial Prescribing Survey. Sydney: ACSQHC; 2018.

6. National Centre for Antimicrobial Stewardship, Australian Commission on Safety and Quality in Health Care. Antimicrobial Prescribing and Infections in Australian Aged Care Homes: Results of the 2018 Aged Care National Antimicrobial Prescribing Survey Sydney: ACSQHC; 2019 [cited 2019 November]. Available from: <https://www.safetyandquality.gov.au/publications-and-resources/resource-library/2018-aged-care-national-antimicrobial-prescribing-survey-report>.

7. National Centre for Antimicrobial Stewardship, Australian Commission on Safety and Quality in Health Care. 2019 Aged Care National Antimicrobial Prescribing Survey Report. Sydney: ACSQHC; 2020.

8. Barney GR, Felsen CB, Dumyati GK. One-day point prevalence as a method for estimating antibiotic use in nursing homes. Infection control and hospital epidemiology. 2019;40(2):221-3.

9. Benoit SR, Nsa W, Richards CL, Bratzler DW, Shefer AM, Steele LM, et al. Factors associated with antimicrobial use in nursing homes: a multilevel model. Journal of the American Geriatrics Society. 2008;56(11):2039-44.

10. Blix HS, Roed J, Sti MO. Large variation in antibacterial use among Norwegian nursing homes. Scandinavian Journal of Infectious Diseases. 2007;39(6-7):536-41.

11. Blix HS, Bergman J, Schjott J. How are antibacterials used in nursing homes? Results from a point-prevalence prescription study in 44 Norwegian nursing homes. Pharmacoepidemiology & Drug Safety. 2010;19(10):1025-30.

12. Boivin Y, Talon D, Leroy J, Floret N, Gbaguidi-Haore H, Bertrand X. Antibiotic prescription in nursing homes for dependent elderly people: a cross-sectional study in Franche-Comte. Medecine et Maladies Infectieuses. 2013;43(4):163-9.

13. Cowan RU, Kishan D, Walton AL, Sneath E, Cheah T, Butwilowsky J, et al. Cleaning, resistant bacteria, and antibiotic prescribing in residential aged care facilities. American Journal of Infection Control. 2016;44(3):e19-21.

14. Daneman N, Gruneir A, Newman A, Fischer HD, Bronskill SE, Rochon PA, et al. Antibiotic use in long-term care facilities. Journal of Antimicrobial Chemotherapy. 2011;66(12):2856-63.

15. Daneman N, Gruneir A, Bronskill SE, Newman A, Fischer HD, Rochon PA, et al. Prolonged antibiotic treatment in long-term care: role of the prescriber. JAMA Internal Medicine. 2013;173(8):673-82.

16. Daneman N, Bronskill SE, Gruneir A, Newman AM, Fischer HD, Rochon PA, et al. Variability in Antibiotic Use Across Nursing Homes and the Risk of Antibiotic-Related Adverse Outcomes for Individual Residents. JAMA Internal Medicine. 2015;175(8):1331-9.

17. Daneman N, Campitelli MA, Giannakeas V, Morris AM, Bell CM, Maxwell CJ, et al. Influences on the start, selection and duration of treatment with antibiotics in long-term care facilities. CMAJ Canadian Medical Association Journal. 2017;189(25):E851-E60.

18. Drinka PJ, Gauerke C, Le D. Antimicrobial use and methicillin-resistant Staphylococcus aureus in a large nursing home. Journal of the American Medical Directors Association. 2004;5(4):256-8.

19. Eikelenboom-Boskamp A, Saris K, van Loosbroek M, Drabbe MIJ, de Jongh F, de Jong JWD, et al. Prevalence of healthcare-associated infections in Dutch nursing homes: follow-up 2010-2017. The Journal of hospital infection. 2019;101(1):49-52.

20. Eriksen HM, Saether AR, Viktil KK, Andberg L, Munkerud MW, Willoch K, et al. Use of antibiotics in nursing homes--surveillance with different methods. Tidsskrift for Den Norske Laegeforening. 2013;133(19):2052-6.

21. Latour K, Catry B, Broex E, Vankerckhoven V, Muller A, Stroobants R, et al. Indications for antimicrobial prescribing in European nursing homes: results from a point prevalence survey. Pharmacoepidemiology & Drug Safety. 2012;21(9):937-44.

22. McClean P, Hughes C, Tunney M, Goossens H, Jans B, European Surveillance of Antimicrobial Consumption Nursing Home Project G. Antimicrobial prescribing in European nursing homes. Journal of Antimicrobial Chemotherapy. 2011;66(7):1609-16.

23. Rummukainen ML, Karki T, Kanerva M, Haapasaari M, Ollgren J, Lyytikainen O. Antimicrobial prescribing in nursing homes in Finland: results of three point prevalence surveys. Infection. 2013;41(2):355-60.

24. McClean P, Tunney M, Gilpin D, Parsons C, Hughes C. Antimicrobial prescribing in residential homes. Journal of Antimicrobial Chemotherapy. 2012;67(7):1781-90.

25. Eure T, LaPlace LL, Melchreit R, Maloney M, Lynfield R, Whitten T, et al. Measuring Antibiotic Appropriateness for Urinary Tract Infections in Nursing Home Residents. Infection Control & Hospital Epidemiology. 2017;38(8):998-1001.

26. Cotter M, Donlon S, Roche F, Byrne H, Fitzpatrick F. Healthcare-associated infection in Irish long-term care facilities: results from the First National Prevalence Study. The Journal of hospital infection. 2012;80(3):212-6.

27. Fagan M, Maehlen M, Lindbaek M, Berild D. Antibiotic prescribing in nursing homes in an area with low prevalence of antibiotic resistance: compliance with national guidelines. Scandinavian Journal of Primary Health Care. 2012;30(1):10-5.

28. Felsen CB, Dodds Ashley ES, Barney GR, Nelson DL, Nicholas JA, Yang H, et al. Reducing Fluoroquinolone Use and Clostridioides difficile Infections in Community Nursing Homes Through Hospital-Nursing Home Collaboration. J Am Med Dir Assoc. 2020;21(1):55-61.e2.

29. Fleet E, Gopal Rao G, Patel B, Cookson B, Charlett A, Bowman C, et al. Impact of implementation of a novel antimicrobial stewardship tool on antibiotic use in nursing homes: a prospective cluster randomized control pilot study. Journal of Antimicrobial Chemotherapy. 2014;69(8):2265-73.

30. Gillespie D, Hood K, Bayer A, Carter B, Duncan D, Espinasse A, et al. Antibiotic prescribing and associated diarrhoea: a prospective cohort study of care home residents. Age & Ageing. 2015;44(5):853-60.

31. Eilers R, Veldman-Ariesen MJ, Haenen A, van Benthem BH. Prevalence and determinants associated with healthcare-associated infections in long-term care facilities (HALT) in the Netherlands, May to June 2010. Euro surveillance : bulletin Europeen sur les maladies transmissibles = European communicable disease bulletin. 2012;17(34).

32. Furmenti MF, Rossello P, Bianco S, Olivero E, Thomas R, Emelurumonye IN, et al. Healthcare-associated infections and antimicrobial use in long-term care facilities (HALT3): an overview of the Italian situation. The Journal of hospital infection. 2019;102(4):425-30.

33. Ricchizzi E, Latour K, Karki T, Buttazzi R, Jans B, Moro ML, et al. Antimicrobial use in European long-term care facilities: results from the third point prevalence survey of healthcare-associated infections and antimicrobial use, 2016 to 2017. Euro surveillance : bulletin Europeen sur les maladies transmissibles = European communicable disease bulletin. 2018;23(46).

34. Tandan M, O'Connor R, Burns K, Murphy H, Hennessy S, Roche F, et al. A comparative analysis of prophylactic antimicrobial use in long-term care facilities in Ireland, 2013 and 2016. Euro surveillance : bulletin Europeen sur les maladies transmissibles = European communicable disease bulletin. 2019;24(11).

35. European Centre for Disease Prevention and Control. Point prevalence survey of healthcare-associated infections and antimicrobial use in European long-term care facilities. April-May 2013. Stockholm: ECDC; 2014.

36. Szabo R, Borocz K. Antimicrobial use in Hungarian long-term care facilities: high proportion of quinolone antibacterials. Archives of Gerontology & Geriatrics. 2014;59(1):190-3.

37. Tandan M, Burns K, Murphy H, Hennessy S, Cormican M, Vellinga A. Antimicrobial prescribing and infections in long-term care facilities (LTCF): a multilevel analysis of the HALT 2016 study, Ireland, 2017. Euro surveillance : bulletin Europeen sur les maladies transmissibles = European communicable disease bulletin. 2018;23(46).

38. European Centre for Disease Prevention and Control. Point prevalence survey of healthcare-associated infections and antimicrobial use in European long-term care facilities. May–September 2010. Stockholm: ECDC; 2014.

39. Moro ML, Ricchizzi E, Morsillo F, Marchi M, Puro V, Zotti CM, et al. Infections and antimicrobial resistance in long term care facilities: a national prevalence study. Annali di Igiene. 2013;25(2):109-18.

40. Heudorf U, Boehlcke K, Schade M. Healthcare-associated infections in long-term care facilities (HALT) in Frankfurt am Main, Germany, January to March 2011. Euro Surveillance: Bulletin Europeen sur les Maladies Transmissibles = European Communicable Disease Bulletin. 2012;17(35):30.

41. Ishikane M, Kusama Y, Tanaka C, Hayakawa K, Kuwahara T, Ohmagari N. Epidemiology of Antimicrobial Use among Nursing Homes in Japan, 2016: a Pilot Study. Jpn J Infect Dis. 2020;73(4):293-5.

42. Jump RL, Olds DM, Seifi N, Kypriotakis G, Jury LA, Peron EP, et al. Effective antimicrobial stewardship in a long-term care facility through an infectious disease consultation service: keeping a LID on antibiotic use. Infection Control & Hospital Epidemiology. 2012;33(12):1185-92.

43. Katz PR, Beam TR, Jr., Brand F, Boyce K. Antibiotic use in the nursing home. Physician practice patterns. Archives of Internal Medicine. 1990;150(7):1465-8.

44. Kabbani S, Palms DL, Bartoces M, Marek J, Stone ND, Hicks LA, et al. Potential utility of pharmacy data to measure antibiotic use in nursing homes. Infection control and hospital epidemiology. 2019;40(7):819-20.

45. Lee YL, Thrupp LD, Friis RH, Fine M, Maleki P, Cesario TC. Nosocomial infection and antibiotic utilization in geriatric patients: a pilot prospective surveillance program in skilled nursing facilities. Gerontology. 1992;38(4):223-32.

46. Lee YL, Thrupp LD, Lee R, Nothvogel S, Farsad N, Cesario T. Infection surveillance and antibiotic utilization in a community-based skilled nursing facility. Aging-Clinical & Experimental Research. 1996;8(2):113-22.

47. Loeb M, Brazil K, Lohfeld L, McGeer A, Simor A, Stevenson K, et al. Effect of a multifaceted intervention on number of antimicrobial prescriptions for suspected urinary tract infections in residents of nursing homes: cluster randomised controlled trial. BMJ. 2005;331(7518):669.

48. Marquet A, Thibaut S, LePabic E, Huon JF, Ballereau F. Three years of antibiotic consumption evaluation in French nursing homes. Medecine et Maladies Infectieuses. 2015;45(8):313-7.

49. Marra F, McCabe M, Sharma P, Zhao B, Mill C, Leung V, et al. Utilization of Antibiotics in Long-Term Care Facilities in British Columbia, Canada. Journal of the American Medical Directors Association. 2017;18(12):1098.e1-.e11.

50. Mayne S, Sundvall PD, Gunnarsson R. Confusion Strongly Associated with Antibiotic Prescribing Due to Suspected Urinary Tract Infections in Nursing Homes. Journal of the American Geriatrics Society. 2018;66(2):274-81.

51. Monette J, Miller MA, Monette M, Laurier C, Boivin JF, Sourial N, et al. Effect of an educational intervention on optimizing antibiotic prescribing in long-term care facilities. Journal of the American Geriatrics Society. 2007;55(8):1231-5.

52. Montgomery P, Semenchuk M, Nicolle LE. Antimicrobial use in nursing homes in Manitoba. Journal of Geriatric Drug Therapy. 1995;9(3):55-74.

53. Moro ML, Mongardi M, Marchi M, Taroni F. Prevalence of long-term care acquired infections in nursing and residential homes in the Emilia-Romagna Region. Infection. 2007;35(4):250-5.

54. Mylotte JM. Antimicrobial prescribing in long-term care facilities: prospective evaluation of potential antimicrobial use and cost indicators. American Journal of Infection Control. 1999;27(1):10-9.

55. Mylotte JM, Keagle J. Benchmarks for antibiotic use and cost in long-term care. Journal of the American Geriatrics Society. 2005;53(7):1117-22.

56. Natsch S, Hekster YA, de Jong R, Heerdink ER, Herings RM, van der Meer JW. Application of the ATC/DDD methodology to monitor antibiotic drug use. European Journal of Clinical Microbiology & Infectious Diseases. 1998;17(1):20-4.

57. Olsho LE, Bertrand RM, Edwards AS, Hadden LS, Morefield GB, Hurd D, et al. Does adherence to the Loeb minimum criteria reduce antibiotic prescribing rates in nursing homes? Journal of the American Medical Directors Association. 2013;14(4):309.e1-7.

58. Pakyz AL, Dwyer LL. Prevalence of antimicrobial use among United States nursing home residents: results from a national survey. Infection Control & Hospital Epidemiology. 2010;31(6):661-2.

59. Pluss-Suard C, Niquille A, Hequet D, Krahenbuhl S, Pichon R, Zanetti G, et al. Decrease in Antibacterial Use and Facility-Level Variability After the Introduction of Guidelines and Implementation of Physician-Pharmacist-Nurse Quality Circles in Swiss Long-term Care Facilities. J Am Med Dir Assoc. 2020;21(1):78-83.

60. Raban MZ, Lind KE, Day RO, Gray L, Georgiou A, Westbrook JI. Trends, determinants and differences in antibiotic use in 68 residential aged care homes in Australia, 2014-2017: a longitudinal analysis of electronic health record data. BMC Health Serv Res. 2020;20(1):883.

61. Rahme CL, Jacoby HM, Avery LM. Impact of a hospital's antibiotic stewardship team on fluoroquinolone use at a long-term care facility. Annals of Long-Term Care. 2016;24(6):13-20.

62. Roukens M, Verhoef L, Stobberingh E, Natsch S. Surveillance of antimicrobial use in Dutch long-term care facilities. Journal of Antimicrobial Chemotherapy. 2017;72(5):1516-20.

63. Rummukainen M, Jakobsson A, Karppi P, Kautiainen H, Lyytikainen O. Promoting hand hygiene and prudent use of antimicrobials in long-term care facilities. American Journal of Infection Control. 2009;37(2):168-71.

64. Rummukainen ML, Makela M, Noro A, Finne-Soveri H, Lyytikainen O. Assessing prevalence of antimicrobial use and infections using the minimal data set in Finnish long-term care facilities. American Journal of Infection Control. 2013;41(4):e35-7.

65. Saxena FE, Bronskill SE, Brown KA, Campitelli MA, Garber G, Langford B, et al. The Association of Resident Communication Abilities and Antibiotic Use in Long-Term Care. Journal of the American Geriatrics Society. 2019;67(6):1164-73.

66. Selcuk A, Teng CB, Chan SY, Yap KZ. Antimicrobial use and drug-drug interactions among nursing home residents in Singapore: a multicentre prevalence study. International Journal of Clinical Pharmacy. 2018.

67. Selcuk A, Yap KZ, Wong CL, Yang JX, Yong PC, Chan SY, et al. A Point Prevalence Study of Antimicrobial Use and Practice Among Nursing Homes in Singapore. Drugs & aging. 2019;36(6):559-70.

68. Sloane PD, Zimmerman S, Reed D, Beeber AS, Chisholm L, Kistler C, et al. Antibiotic prescribing in 4 assisted-living communities: incidence and potential for improvement. Infection Control & Hospital Epidemiology. 2014;35 Suppl 3:S62-8.

69. Sloane PD, Zimmerman S, Ward K, Kistler CE, Paone D, Weber DJ, et al. A 2-Year Pragmatic Trial of Antibiotic Stewardship in 27 Community Nursing Homes. Journal of the American Geriatrics Society. 2020.

70. Sluggett JK, Moldovan M, Lynn DJ, Papanicolas LE, Crotty M, Whitehead C, et al. National Trends in Antibiotic Use in Australian Residential Aged Care Facilities, 2005-2016. Clin Infect Dis. 2020.

71. Smith M, Atkins S, Worth L, Richards M, Bennett N. Infections and antimicrobial use in Australian residential aged care facilities: a comparison between local and international prevalence and practices. Australian Health Review. 2013;37(4):529-34.

72. Smith CM, Williams H, Jhass A, Patel S, Crayton E, Lorencatto F, et al. Antibiotic prescribing in UK care homes 2016-2017: retrospective cohort study of linked data. BMC Health Serv Res. 2020;20(1):555.

73. Song S, Wilson BM, Bej T, Gravenstein S, Carter RR, Marek J, et al. Antibiotic Use Among Residents Receiving Skilled Nursing Care in 29 U.S. Nursing Homes. Journal of the American Geriatrics Society. 2021;69(2):399-406.

74. Stepan D, Usaj L, Petek Ster M, Smolinger Galun M, Smole H, Beovic B. Antimicrobial prescribing in long-term care facilities: a nationwide point-prevalence study, Slovenia, 2016. Euro surveillance : bulletin Europeen sur les maladies transmissibles = European communicable disease bulletin. 2018;23(46).

75. Stuart RL, Wilson J, Bellaard-Smith E, Brown R, Wright L, Vandergraaf S, et al. Antibiotic use and misuse in residential aged care facilities. Internal Medicine Journal. 2012;42(10):1145-9.

76. Stuart RL, Orr E, Kotsanas D, Gillespie EE. A nurse-led antimicrobial stewardship intervention in two residential aged care facilities. Healthcare Infection. 2015;20(1):4-6.

77. Sundvall PD, Stuart B, Davis M, Roderick P, Moore M. Antibiotic use in the care home setting: a retrospective cohort study analysing routine data. BMC Geriatrics. 2015;15:71.

78. Takito S, Kusama Y, Fukuda H, Kutsuna S. Pharmacist-supported antimicrobial stewardship in a retirement home. J Infect Chemother. 2020;26(8):858-61.

79. Taxis K, Kochen S, Wouters H, Boersma F, Jan Gerard M, Mulder H, et al. Cross-national comparison of medication use in Australian and Dutch nursing homes. 2017:320-3 LID - 10.1093/ageing/afw218 [doi].

80. Temime L, Cohen N, Ait-Bouziad K, Denormandie P, Dab W, Hocine MN. Impact of a multicomponent hand hygiene-related intervention on the infectious risk in nursing homes: A cluster randomized trial. American Journal of Infection Control. 2018;46(2):173-9.

81. Thompson ND, LaPlace L, Epstein L, Thompson D, Dumyati G, Concannon C, et al. Prevalence of Antimicrobial Use and Opportunities to Improve Prescribing Practices in U.S. Nursing Homes. Journal of the American Medical Directors Association. 2016;17(12):1151-3.

82. Thompson ND, Stone ND, Brown CJ, Penna AR, Eure TR, Bamberg WM, et al. Antimicrobial Use in a Cohort of US Nursing Homes, 2017. Jama. 2021;325(13):1286-95.

83. Thornley T, Ashiru-Oredope D, Normington A, Beech E, Howard P. Antibiotic prescribing for residents in long-term-care facilities across the UK. Journal of Antimicrobial Chemotherapy. 2019;74(5):1447-51.

84. Thornley T, Ashiru-Oredope D, Beech E, Howard P, Kirkdale CL, Elliott H, et al. Antimicrobial use in UK long-term care facilities: Results of a point prevalence survey. Journal of Antimicrobial Chemotherapy. 2019;74(7):2083-90.

85. van Buul LW, van der Steen JT, Achterberg WP, Schellevis FG, Essink RT, de Greeff SC, et al. Effect of tailored antibiotic stewardship programmes on the appropriateness of antibiotic prescribing in nursing homes. Journal of Antimicrobial Chemotherapy. 2015;70(7):2153-62.

86. Warren JW, Palumbo FB, Fitterman L, Speedie SM. Incidence and characteristics of antibiotic use in aged nursing home patients. Journal of the American Geriatrics Society. 1991;39(10):963-72.

87. Wu LDY, Walker SAN, Elligsen M, Paimay L, Simor A, Daneman N. Antibiotic use and need for antimicrobial stewardship in long-term care. Canadian Journal of Hospital Pharmacy. 2015;68(6):445-9.

88. Zimmerman S, Sloane PD, Bertrand R, Olsho LE, Beeber A, Kistler C, et al. Successfully reducing antibiotic prescribing in nursing homes. Journal of the American Geriatrics Society. 2014;62(5):907-12.
