# Supplementary material for: Temporal and regional trends of antibiotic use in long-term aged care facilities across 39 countries, 1985-2019: Systematic review and meta-analysis
Source: PLoS One. 2021 Aug 23;16(8):e0256501. doi: 10.1371/journal.pone.0256501 (PMC8382177; doi:10.1371/journal.pone.0256501)
Supplement: S5 File — (DOCX) [file pone.0256501.s005.docx]

**S5 File: Point prevalence meta-analysis results, reported resident characteristics, and resident eligibility**

Table A: Meta-analysis results of point prevalence estimates of antibiotic use by region and overall

| **Region** | **Study name** | **Point prevalence** | **95% CI** | **Country** | **Year** |
| --- | --- | --- | --- | --- | --- |
| Singapore | Selcuk, 2019 | 2.97 | 1.94, 4.51 | Singapore | 2008 |
|  | Selcuk, 2019 | 2.33 | 1.72, 3.15 | Singapore | 2017 |
|  | Overall | 2.61 | 1.43, 4.74 |  |  |
| Australia | Smith, 2013 | 8.32 | 6.55, 10.51 | Australia | 2011 |
|  | Stuart, 2012 | 8.95 | 6.02, 13.11 | Australia | 2011 |
|  | acNAPS, 2016 | 7.91 | 7.32, 8.53 | Australia | 2015 |
|  | acNAPS, 2017 | 7.50 | 7.07, 7.96 | Australia | 2016 |
|  | acNAPS, 2018 | 6.70 | 6.27, 7.16 | Australia | 2017 |
|  | acNAPS, 2019 | 6.70 | 6.36, 7.05 | Australia | 2018 |
|  | acNAPS, 2020 | 5.47 | 5.22, 5.72 | Australia | 2019 |
|  | OVERALL | 7.22 | 5.42, 9.57 |  |  |
| British Isles | ESAC-1 (4) | 11.7 | 8.17, 16.6 | England | 2009 |
|  | HALT-1 (23) | 12.7 | 9.94, 16.0 | England | 2010 |
|  | Fleet, 2014 (1) | 6.51 | 5.41, 7.82 | England | 2010 |
|  | Fleet, 2014 (2) | 5.53 | 4.51, 6.76 | England | 2011 |
|  | HALT-2 (14) | 9.05 | 6.62, 12.2 | England | 2013 |
|  | HALT-3 (24) | 6.25 | 5.87, 6.65 | England | 2017 |
|  | ESAC-1 (9) | 10.8 | 9.42, 12.4 | Ireland | 2009 |
|  | HALT-1 (14) | 11.3 | 10.3, 12.4 | Ireland | 2010 |
|  | HALT-2 (17) | 9.80 | 9.21, 10.4 | Ireland | 2013 |
|  | HALT-3 (25) | 9.77 | 9.20, 10,36 | Ireland | 2017 |
|  | ESAC-1 (15) | 13.0 | 11.02, 15.26 | N Ireland | 2009 |
|  | ESAC-2 (2) | 9.40 | 7.29, 12.05 | N Ireland | 2010 |
|  | HALT-1 (24) | 8.72 | 6.77, 11.17 | N Ireland | 2010 |
|  | ESAC-3 (2) | 9.17 | 7.07, 11.81 | N Ireland | 2011 |
|  | HALT-2 (15) | 10.6 | 9.02, 12.46 | N Ireland | 2013 |
|  | HALT-3 (21) | 10.3 | 9.22, 11.56 | N Ireland | 2017 |
|  | ESAC-1 (19) | 9.66 | 7.97, 11.66 | Scotland | 2009 |
|  | HALT-1 (25) | 7.39 | 6.69, 8.16 | Scotland | 2010 |
|  | HALT-3 (22) | 6.43 | 5.46, 7.55 | Scotland | 2017 |
|  | HALT-1 (26) | 6.53 | 5.01, 8.47 | Wales | 2010 |
|  | HALT-2 (16) | 7.47 | 6.47, 8.62 | Wales | 2013 |
|  | HALT-3 (23) | 10.14 | 8.39, 12.21 | Wales | 2017 |
|  | OVERALL | 8.95 | 7.63, 10.5 |  |  |
| Eastern Europe | HALT-1 (3) | 2.50 | 1.04, 5.86 | Bulgaria | 2010 |
|  | ESAC-1 (1) | 1.40 | 0.88, 2.20 | Croatia | 2009 |
|  | HALT-1 (4) | 1.83 | 0.99, 3.37 | Croatia | 2010 |
|  | HALT-2 (2) | 1.79 | 0.90, 3.54 | Croatia | 2013 |
|  | HALT-3 (4) | 1.99 | 1.41, 2.80 | Croatia | 2016 |
|  | ESAC-1 (2) | 7.53 | 5.78, 9.74 | Czech Republic | 2009 |
|  | HALT-1 (6) | 2.91 | 1.79, 4.70 | Czech Republic | 2010 |
|  | HALT-2 (3) | 11.33 | 9.13, 13.98 | Czech Republic | 2013 |
|  | HALT-1 (8) | 0.77 | 0.34, 1.69 | Estonia | 2010 |
|  | HALT-3 (26) | 8.84 | 6.09, 12.67 | Former Yugoslav | 2016 |
|  | HALT-1 (13) | 1.55 | 1.24, 1.94 | Hungary | 2010 |
|  | Szabo, 2014 | 1.32 | 1.13, 1.54 | Hungary | 2013 |
|  | HALT-3 (11) | 0.93 | 0.73, 1.17 | Hungary | 2016 |
|  | ESAC-1 (11) | 1.00 | 0.57, 1.76 | Latvia | 2009 |
|  | ESAC-1 (12) | 2.38 | 0.77, 7.12 | Lithuania | 2009 |
|  | HALT-1 (15) | 0.91 | 0.61, 1.37 | Lithuania | 2010 |
|  | HALT-3 (12) | 0.73 | 0.49, 1.07 | Lithuania | 2016 |
|  | ESAC-1 (17) | 5.06 | 3.65, 6.96 | Poland | 2009 |
|  | HALT-1 (19) | 1.28 | 0.48, 3.35 | Poland | 2010 |
|  | HALT-3 (17) | 3.20 | 2.55, 4.01 | Poland | 2016 |
|  | ESAC-1 (18) | 1.26 | 0.83, 1.91 | Russia Federation | 2009 |
|  | HALT-3 (2) | 4.88 | 3.78, 6.28 | Serbia | 2016 |
|  | HALT-3(18) | 2.22 | 1.85, 2.66 | Slovakia | 2016 |
|  | ESAC-1 (20) | 3.59 | 2.74, 4.69 | Slovenia | 2009 |
|  | HALT-1 (20) | 2.29 | 1.63, 3.22 | Slovenia | 2010 |
|  | HALT-2 (12) | 1.49 | 0.48, 4.50 | Slovenia | 2013 |
|  | Stepan, 2018 | 2.43 | 2.18, 2.71 | Slovenia | 2016 |
|  | OVERALL | 2.43 | 1.93, 2.72 |  |  |
| North America | Daneman, 2011 | 5.86 | 5.63, 6.10 | Canada | 2009 |
|  | Warren, 1991 | 7.80 | 7.03, 8.66 | US | 1985 |
|  | Thompson, 2016 | 11.08 | 9.47, 12.9 | US | 2014 |
|  | Thompson, 2021 | 8.23 | 7.81, 8.68 | US | 2017 |
|  | OVERALL | 8.03 | 5.53, 11.5 |  |  |
| Northern Europe | ESAC-1 (3) | 9.72 | 6.92, 13.49 | Denmark | 2009 |
|  | HALT-1 (7) | 10.86 | 7.86, 14.82 | Denmark | 2010 |
|  | HALT-2 (4) | 11.30 | 9.67, 13.17 | Denmark | 2013 |
|  | HALT-3 (6) | 10.46 | 9.47, 11.54 | Denmark | 2016 |
|  | Rummukainen, 2009 (1) | 16.63 | 14.64, 18.82 | Finland | 2005 |
|  | Rummukainen, 2009 (2) | 12.30 | 10.57, 14.28 | Finland | 2006 |
|  | Rummukainen, 2009 (3) | 12.22 | 10.48, 14.20 | Finland | 2007 |
|  | Rummukainen, 2009 (4) | 11.31 | 9.61, 13.27 | Finland | 2008 |
|  | ESAC-1 (5) | 12.43 | 10.93, 14.11 | Finland | 2009 |
|  | ESAC-1 (6) | 17.29 | 15.57, 19.16 | Finland | 2009 |
|  | ESAC-2 (1) | 9.74 | 8.60, 11.02 | Finland | 2010 |
|  | HALT-1 (9) | 9.78 | 8.64, 11.1 | Finland | 2010 |
|  | ESAC-3 (1) | 15.8 | 15.1, 16.4 | Finland | 2011 |
|  | HALT-2 (5) | 6.64 | 4.71, 9.28 | Finland | 2013 |
|  | HALT-3 (7) | 6.66 | 6.05, 7.33 | Finland | 2016 |
|  | Blix, 2010 | 15.2 | 13.46, 17.13 | Norway | 2006 |
|  | ESAC-1 (16) | 9.15 | 7.04, 11.82 | Norway | 2009 |
|  | HALT-2 (11) | 8.30 | 6.95, 9.88 | Norway | 2013 |
|  | Alberg, 2017 | 7.20 | 6.88, 7.54 | Norway | 2016 |
|  | HALT-3 (16) | 6.91 | 5.97, 7.98 | Norway | 2016 |
|  | ESAC-1 (21) | 3.35 | 2.09, 5.32 | Sweden | 2009 |
|  | HALT-1 (22) | 3.56 | 1.93, 6.69 | Sweden | 2010 |
|  | HALT-2 (13) | 2.72 | 2.00, 3.71 | Sweden | 2013 |
|  | HALT-3 (20) | 3.27 | 2.74, 3.91 | Sweden | 2016 |
|  | OVERALL | 9.02 | 7.73, 10.5 |  |  |
| Southern Europe | HALT-1 (5) | 1.56 | 0.22, 10.27 | Cyprus | 2010 |
|  | HALT-3 (5) | 9.29 | 6.54, 13.06 | Cyprus | 2016 |
|  | HALT-1 (12) | 2.83 | 1.79, 4.45 | Greece | 2010 |
|  | HALT-2 (7) | 7.44 | 5.00, 10.95 | Greece | 2013 |
|  | HALT-3 (10) | 6.03 | 4.59, 7.90 | Greece | 2016 |
|  | Moro, 2007 | 12.2 | 10.76, 13.69 | Italy | 2002 |
|  | ESAC-1 (10) | 5.21 | 4.45, 6.10 | Italy | 2009 |
|  | Moro, 2013 | 4.72 | 4.30, 5.17 | Italy | 2010 |
|  | HALT-2 (8) | 3.95 | 3.67, 4.24 | Italy | 2013 |
|  | HALT-3 (1) | 4.57 | 4.31, 4.84 | Italy | 2017 |
|  | ESAC-1 (13) | 2.81 | 1.47, 5.32 | Malta | 2009 |
|  | HALT-1 (17) | 2.83 | 1.68, 4.72 | Malta | 2010 |
|  | HALT-2 (9) | 3.21 | 2.44, 4.21 | Malta | 2013 |
|  | HALT-3 (14) | 2.66 | 2.09, 3.37 | Malta | 2016 |
|  | HALT-1 (21) | 0.79 | 0.11, 5.51 | Spain | 2010 |
|  | HALT-3 (19) | 10.53 | 9.82, 11.28 | Spain | 2016 |
|  | OVERALL | 4.92 | 3.97, 6.08 |  |  |
| W Europe | HALT-1 (1) | 1.75 | 0.84, 3.62 | Austria | 2010 |
|  | HALT-3 (1) | 3.24 | 2.56, 4.10 | Austria | 2016 |
|  | ESAC-1 (22) | 5.64 | 5.25, 6.07 | Belgium | 2009 |
|  | HALT-1 (2) | 4.32 | 3.97, 4.70 | Belgium | 2010 |
|  | HALT-2 (1) | 5.06 | 4.62, 5.54 | Belgium | 2013 |
|  | HALT-3 (3) | 5.87 | 5.39, 6.40 | Belgium | 2016 |
|  | ESAC-1 (7) | 5.16 | 4.31, 6.16 | France | 2009 |
|  | HALT-1 (10) | 3.07 | 2.67, 3.53 | France | 2010 |
|  | Boivin, 2013 | 2.76 | 2.15, 3.53 | France | 2012 |
|  | HALT-3 (8) | 2.69 | 2.33, 3.20 | France | 2016 |
|  | ESAC-1 (8) | 1.18 | 0.49, 2.79 | Germany | 2009 |
|  | HALT-1 (11) | 1.15 | 0.92, 1.45 | Germany | 2010 |
|  | Heudorf, 2012 | 2.40 | 1.96, 2.94 | Germany | 2011 |
|  | HALT-2 (6) | 1.86 | 1.67, 2.08 | Germany | 2013 |
|  | HALT-3 (9) | 1.27 | 1.03, 1.57 | Germany | 2016 |
|  | HALT-1 (16) | 4.53 | 3.03, 6.72 | Luxembourg | 2010 |
|  | HALT-3 (13) | 2.60 | 1.93, 3.50 | Luxembourg | 2016 |
|  | ESAC-1 (14) | 4.78 | 3.43, 6.61 | Netherlands | 2009 |
|  | HALT-1 (18) | 3.50 | 2.66, 4.59 | Netherlands | 2010 |
|  | HALT-2 (10) | 6.10 | 4.47, 8.27 | Netherlands | 2013 |
|  | HALT-3 (15) | 4.44 | 3.88, 5.08 | Netherlands | 2016 |
|  | OVERALL | 3.22 | 2.70, 3.84 |  |  |
| **Overall (all regions)** |  | **5.17** | **3.33, 7.93** |  |  |

Table B: Resident characteristics reported in studies included in point prevalence meta-analysis by region

| **Region / Study name** | **Country** | **Year** | **Mean age** | **Median age** | **% >85 years** | **% female** | **% with urinary catheter** | **% with indwelling medical devices** | **% with dementia** | **% with cognitive impairment or disorientation** | **% with wounds (other than pressure sores)** | **% with pressure sores** |
| --- | --- | --- | --- | --- | --- | --- | --- | --- | --- | --- | --- | --- |
| **Singapore** | | | | | | | | | | | | |
| Selcuk, 2019 | Singapore | 2008 | - | - | - | - | - | - | - | - | - | - |
| Selcuk, 2019 | Singapore | 2017 | - | - | - | - | - | - | - | - | - | - |
| **Australia** | | | | | | | | | | | | |
| Smith, 2013 | Australia | 2011 | - | - | 52.7 | - | 6 | - | - | - | 16.0 | 8.7 |
| Stuart, 2012 | Australia | 2011 | - | - | 28 | 51 | 3 | - | - | 80 | - | 5 |
| acNAPS, 2016 | Australia | 2015 | - | - | 47.7 | 65.6 | 4.3 | - | - | - | - | - |
| acNAPS, 2017 | Australia | 2016 | - | - | 54.3 | 67.1 | 3.8 | - | - | - | - | - |
| acNAPS, 2018 | Australia | 2017 | - | - | 57.9 | 66.0 | 3.8 | - | - | - | - | - |
| acNAPS, 2019 | Australia | 2018 | - | - | 59.4 | 67.3 | 3.8 | - | - | - | - | - |
| acNAPS, 2020 | Australia | 2019 | - | - | - | - | - | - | - | - | - | - |
| **British Isles** | | | | | | | | | | | | |
| ESAC-1 (15) | N Ireland | 2009 | 83 | - | - | - | 7.6 | - | - | 61.3 | - | - |
| HALT-1 | England | 2010 | - | - | 40.9 | 68.3 | 11.9 | - | - | 63.5 | 13.3 | 5.8 |
| Fleet, 2014 (1) | England | 2010 | - | - | IG: 46.7  CG: 44.1 | IG: 65.9  CG: 67.6 | - | IG: 11.2  CG: 6.6 | - | - | IG: 10.6  CG: 9.3 | - |
| Fleet, 2014 (2) | England | 2011 | - | - | IG: 44.6  CG: 44.1 | IG: 66.5  CG: 66.8 | - | IG: 13.4  CG: 10.5 | - | - | IG: 8.6  CG: 10.5 | - |
| HALT-2 (14) | England | 2013 | - | - | 45.6 | 63.8 | - | - | - | - | - | - |
| HALT-3 (24) | England | 2017 | - | - | - | - | - | - | - | - | - | - |
| ESAC-1 (9) | Ireland | 2009 | - | - | - | - | - | - | - | - | - | - |
| HALT-1 | Ireland | 2010 | - | - | 41.5 | 61.9 | 6.6 | - | - | 47.8 | 10.4 | 3.1 |
| HALT-2 (17) | Ireland | 2013 | - | - | 45.2 | 66.2 | - | - | - | - | - | - |
| HALT-3 | Ireland | 2016 | - | - | 39.6 | 62.5 | 13.7 | - | - | - | - | 5.7 |
| ESAC-1 (15) | N Ireland | 2009 | - | - | - | - | 7.6 | - | - | 61.3 | - | - |
| ESAC-2 (2) | N Ireland | 2010 | 84.7 | - | - | 75.2 | 2.1 | - | - | 47.2 | 2.9 | 1.2 |
| HALT-1 (24) | N Ireland | 2010 | - | - | 44.6 | 71.1 | 5.7 | - | - | 49.8 | 6.3 | 3.6 |
| ESAC-3 (2) | N Ireland | 2011 | 84.5 | - | - | 75.6 | 1.9 | - | - | 42.4 | 2.9 | 1.2 |
| HALT-2 (15) | N Ireland | 2013 | - | - | 46.4 | 67.4 | - | - | - | - | - | - |
| HALT-3 (21) | N Ireland | 2017 | - | - | - | - | - | - | - | - | - | - |
| ESAC-1 (19) | Scotland | 2009 | - | - | - | - | - | - | - | - | - | - |
| HALT-1 (25) | Scotland | 2010 | - | - | 44.6 | 71.9 | 8.3 | - | - | 62.4 | 4.5 | 3.5 |
| HALT-3 (22) | Scotland | 2017 | - | - | - | - | - | - | - | - | - | - |
| HALT-1 (26) | Wales | 2010 | - | - | 49.4 | 69.6 | 10.4 | - | - | 51.8 | 3.5 | 4.7 |
| HALT-2 (16) | Wales | 2013 | - | - | 55.5 | 67.6 | - | - | - | - | - | - |
| HALT-3 (23) | Wales | 2017 | - | - | - | - | - | - | - | - | - | - |
| **Eastern Europe** | | | | | | | | | | | | |
| HALT-1 (3) | Bulgaria | 2010 | - | - | 23.0 | 62.4 | - | - | - | - | 4.7 | 9.0 |
| ESAC-1 (1) | Croatia | 2009 | - | - | - | - | - | - | - | - | - | - |
| HALT-1 (4) | Croatia | 2010 | - | - | 37.5 | 73.7 | - | - | - | - | 3.9 | 1.1 |
| HALT-2 (2) | Croatia | 2013 | - | - | 43.1 | 73.1 | - | - | - | - | - | - |
| HALT-3 (4) | Croatia | 2016 | - | - | - | - | - | - | - | - | - | - |
| ESAC-1 (2) | Czech Republic | 2009 | - | - | - | - | - | - | - | - | - | - |
| HALT-1 (6) | Czech Republic | 2010 | - | - | 59.7 | 59.0 | - | - | - | - | 9.9 | 13.6 |
| HALT-2 (3) | Czech Republic | 2013 | - | - | 40.4 | 59.4 | - | - | - | - | - | - |
| HALT-1 (8) | Estonia | 2010 | - | - | - | - | - | - | - | - | - | - |
| HALT-3 (26) | Former Yugoslavia | 2016 | - | - | - | - | - | - | - | - | - | - |
| HALT-1 (13) | Hungary | 2010 | - | - | 26.8 | 68.8 | - | - | - | - | 5.4 | 2.6 |
| Szabo, 2014 | Hungary | 2013 | - | - | - | - | - | - | - | - | - | - |
| HALT-3 (11) | Hungary | 2016 | - | - | - | - | - | - | - | - | - | - |
| ESAC-1 (11) | Latvia | 2009 | - | - | - | - | - | - | - | - | - | - |
| ESAC-1 (12) | Lithuania | 2009 | - | - | - | - | - | - | - | - | - | - |
| HALT-1 (15) | Lithuania | 2010 | - | - | 28.4 | 68.9 | - | - | - | - | - | - |
| HALT-3 (12) | Lithuania | 2016 | - | - | - | - | - | - | - | - | - | - |
| ESAC-1 (17) | Poland | 2009 | - | - | - | - | - | - | - | - | - | - |
| HALT-1 (19) | Poland | 2010 | - | - | 14.8 | 66.6 | - | - | - | - | 1.6 | 2.6 |
| HALT-3 (17) | Poland | 2016 | - | - | - | - | - | - | - | - | - | - |
| ESAC-1 (18) | Russia Federation | 2009 | - | - | - | - | - | - | - | - | - | - |
| HALT-3 (2) | Serbia | 2016 | - | - | - | - | - | - | - | - | - | - |
| HALT-3(18) | Slovakia | 2016 | - | - | - | - | - | - | - | - | - | - |
| ESAC-1 (20) | Slovenia | 2009 | - | - | - | - | - | - | - | - | - | - |
| HALT-1 (20) | Slovenia | 2010 | - | - | 42.8 | 68.0 | - | - | - | - | 5.3 | 3.4 |
| HALT-2 (12) | Slovenia | 2013 | - | - | 48.5 | 77.2 | - | - | - | - | - | - |
| Stepan, 2018 | Slovenia | 2016 | - | - | 63.0* | - | - | - | - | - | - | - |
| **North America** | | | | | | | | | | | | |
| Daneman, 2011 | Canada | 2009 | - | 85 | - | 72.2 | - | 2.8 | 59.5 | - | - | - |
| Warren, 1991 | US | 1985 | - | - | - | - | - | - | - | - | - | - |
| Thompson, 2016 | US | 2014 | - | 85 | - | 70.0 | - | - | - | - | - | - |
| Thompson, 2021 | US | 2017 | 77.6 |  | 36.8 | 62.0 | 7.0 | 5.3 | - | - | 18.6 | 7.3 |
| **Northern Europe** | | | | | | | | | | | | |
| ESAC-1 (3) | Denmark | 2009 | - | - | - | - | - | - | - | - | - | - |
| HALT-1 (7) | Denmark | 2010 | - | - | 51.2 | 67.3 | - | - | - | - | 8.6 | 1.6 |
| HALT-2 (4) | Denmark | 2013 | - | - | 51.0 | 65.7 | - | - | - | - | - | - |
| HALT-3 (6) | Denmark | 2016 | - | - | - | - | - | - | - | - | - | - |
| Rummukainen, 2009 (1) | Finland | 2005 | - | - | - | 71.4 | 1.0 | - | 59.9 | - | - | 2.5 |
| Rummukainen, 2009 (2) | Finland | 2006 | - | - | - | - | - | - | - | - | - | - |
| Rummukainen, 2009 (3) | Finland | 2007 | - | - | - | - | - | - | - | - | - | - |
| Rummukainen, 2009 (4) | Finland | 2008 | - | - | - | - | - | - | - | - | - | - |
| ESAC-1 (5) | Finland | 2009 | - | - | - | - | 2.2 | - | - | 67.6 | 7.3 | - |
| ESAC-1 (6) | Finland | 2009 | - | - | - | 74.2 | 3.1 | - | - | 69.6 | 10.2 | - |
| ESAC-2 (1) | Finland | 2010 | - | - | 49.7 | 50.3 | 3.0 | - | - | 68.0 | 9.5 | - |
| HALT-1 (9) | Finland | 2010 | - | - | 45.8 | 73.8 | - | - | - | - | 5.5 | 3.2 |
| ESAC-3 (1) | Finland | 2011 | - | - | 49.0 | 73.0 | 4.0 | - | - | 79.0 | 2.0 | - |
| HALT-2 (5) | Finland | 2013 | - | - | 48.8 | 73.2 | - | - | - | - | - | - |
| HALT-3 (7) | Finland | 2016 | - | - | - | - | - | - | - | - | - | - |
| Blix, 2010 | Norway | 2006 | - | - | - | - | - | - | - | - | - | - |
| ESAC-1 (16) | Norway | 2009 | - | - | - | - | 5.8 | - | - | 70.7 | - | - |
| HALT-2 (11) | Norway | 2013 | - | - | 58.7 | 33.0 | - | - | - | - | - | - |
| Alberg, 2017 | Norway | 2016 | - | - | - | - | 8.2 | - | - | - | - | - |
| HALT-3 (16) | Norway | 2016 | - | - | - | - | - | - | - | - | - | - |
| ESAC-1 (21) | Sweden | 2009 | - | - | - | - | - | - | - | - | - | - |
| HALT-1 (22) | Sweden | 2010 | - | - | 58.3 | 65.8 | - | - | - | - | - | - |
| HALT-2 (13) | Sweden | 2013 | - | - | 52.4 | 68.8 | - | - | - | - | - | - |
| HALT-3 (20) | Sweden | 2016 | - | - | - | - | - | - | - | - | - | - |
| **Southern Europe** | | | | | | | | | | | | |
| HALT-1 (5) | Cyprus | 2010 | - | - | 50.2 | 69.8 | 6.4 | - | - | - | 1.4 | 0.0 |
| HALT-3 (5) | Cyprus | 2016 | - | - | - | - | - | - | - | - | - | - |
| HALT-1 (12) | Greece | 2010 | - | - | 36.7 | 53.0 | 10.3 | - | - | - | 0.4 | 8.1 |
| HALT-2 (7) | Greece | 2013 | - | - | 47.6 | 8.5 | - | - | - | - | - | - |
| HALT-3 (10) | Greece | 2016 | - | - | - | - | - | - | - | - | - | - |
| Moro, 2007 | Italy | 2002 | - | 85 | - | 75.0 | 8.7 | - | - | 63.1 | - | - |
| ESAC-1 (10) | Italy | 2009 | - | - | - | - | - | - | - | - | - | - |
| Moro, 2013 | Italy | 2010 | - | - | 48.7 | 76.3 | - | - | - | - | - | - |
| HALT-2 (8) | Italy | 2013 | - | - | 50.4 | 72.4 | - | - | - | - | - | - |
| HALT-3 (1) | Italy | 2017 | - | - | 53.9 | 81.7 | 10.1 | - | - | - | 8.4 | 8.5 |
| ESAC-1 (13) | Malta | 2009 | - | - | - | - | - | - | - | - | - | - |
| HALT-1 (17) | Malta | 2010 | - | - | 47.2 | 70.5 | 4.6 | - | - | - | 2.6 | 2.6 |
| HALT-2 (9) | Malta | 2013 | - | - | 49.8 | 79.1 | - | - | - | - | - | - |
| HALT-3 (14) | Malta | 2016 | - | - | - | - | - | - | - | - | - | - |
| HALT-1 (21) | Spain | 2010 | - | - | 42.7 | 64.6 | 12.4 | - | - | - | 9.1 | 18.1 |
| HALT-3 (19) | Spain | 2016 | - | - | - | - | - | - | - | - | - | - |
| **Western Europe** |  |  |  |  |  |  |  |  |  |  |  |  |
| HALT-1 (1) | Austria | 2010 | - | - | 51.4 | 80.1 | 7.1 | - | - | - | 13.4 | 3.3 |
| HALT-3 (1) | Austria | 2016 | - | - | - | - | - | - | - | - | - | - |
| ESAC-1 (22) | Belgium | 2009 | - | - | - | - | - | - | - | - | - | - |
| HALT-1 (2) | Belgium | 2010 | - | - | 53.4 | 74.7 | 2.6 | - | - | - | 8.2 | 3.5 |
| HALT-2 (1) | Belgium | 2013 | - | - | 60.2 | 75.2 | - | - | - | - | - | - |
| HALT-3 (3) | Belgium | 2016 | - | - | - | - | - | - | - | - | - | - |
| ESAC-1 (7) | France | 2009 | - | - | - | - | - | - | - | - | - | - |
| HALT-1 (10) | France | 2010 | - | - | 53.1 | 70.9 | 1.4 | - | - | - | 9.5 | 4.4 |
| Boivin, 2013 | France | 2012 | - | - | - | - | - | - | - | - | - | - |
| HALT-3 (8) | France | 2016 | - | - | - | - | - | - | - | - | - | - |
| ESAC-1 (8) | Germany | 2009 | - | - | - | - | - | - | - | - | - | - |
| HALT-1 (11) | Germany | 2010 | - | - | 49.4 | 73.2 | 10.2 | - | - | - | 5.1 | 3.8 |
| Heudorf, 2012 | Germany | 2011 | - | - | - | 71.5 | 10.1 | - | - | - | 5.3 | 4.2 |
| HALT-2 (6) | Germany | 2013 | - | - | 49.0 | 73.5 | - | - | - | - | - | - |
| HALT-3 (9) | Germany | 2016 | - | - | - | - | - | - | - | - | - | - |
| HALT-1 (16) | Luxembourg | 2010 | - | - | 46.6 | 69.2 | 6.2 | - | - | - | 11.7 | 7.6 |
| HALT-3 (13) | Luxembourg | 2016 | - | - | - | - | - | - | - | - | - | - |
| ESAC-1 (14) | Netherlands | 2009 | - | - | - | - | - | - | - | - | - | - |
| HALT-1 (18) | Netherlands | 2010 | - | - | 38.7 | 68.4 | 11.1 | - | - | - | 7.1 | 5.3 |
| HALT-2 (10) | Netherlands | 2013 | - | - | 40.6 | 63.0 | - | - | - | - | - | - |
| HALT-3 (15) | Netherlands | 2016 | - | - | - | - | - | - | - | - | - | - |
| **Overall for European survey program** | | | | |  |  |  |  |  |  |  |  |
| HALT-2 | 19 countries | 2013 | - | - | 46.5 | 69.3 | 8.8 | - | - | - | 9.4 | 6.0 |

‘-‘ indicates data was not reported. IG is intervention group. CG is control group.

*Percentage of residents ≥ 80 years

Table C: Eligibility criteria for resident inclusion in point prevalence survey as reported in studies included in point prevalence meta-analysis

| **Study name** | **Country** | **Year** | **Eligibility criteria** |
| --- | --- | --- | --- |
| acNAPS, 2016 | Australia | 2015 | All residents present on day of survey. |
| acNAPS, 2017 | Australia | 2016 | All residents present on day of survey. |
| acNAPS, 2018 | Australia | 2017 | All residents present on day of survey. |
| acNAPS, 2019 | Australia | 2018 | All residents present on day of survey. |
| acNAPS, 2020 | Australia | 2019 | All residents present on day of survey. |
| Alberg, 2017 | Norway | 2016 | All residents present on day of survey. |
| Blix, 2010 | Norway | 2006 | All residents present on day of survey. |
| Boivin, 2013 | France | 2012 | Data was obtained by prescription review, though residents who were treated with antibiotics were asked to provide consent to participate. |
| Daneman, 2011 | Canada | 2009 | All elderly Ontarians (≥66 years old) residing in a long-term care facility who had a completed Continuing Care Reporting System Long Term Care assessment were eligible. |
| ESAC-1 | 21 European countries | 2009 | A resident was eligible to be included if he/she had been living full time in the NH for at least 24 hours and was present at 8 AM on the day of the survey. |
| ESAC-2 | N Ireland, Finland | 2010 | A resident was eligible to be included if he/she had been living full time in the NH for at least 24 hours and was present at 8 AM on the day of the survey. |
| ESAC-3 | N Ireland | 2011 | A resident was eligible to be included if he/she had been living full time in the NH for at least 24 hours and was present at 8 AM on the day of the survey. |
| Fleet, 2014 | England | 2010, 2011 | All residents present on day of survey. |
| HALT-1 | 28 European countries | 2010 | A resident was eligible to be included if he/she had been living full time in the NH for at least 24 hours and was present at 8 AM on the day of the survey. |
| HALT-2 | 19 European countries | 2013 | A resident was eligible to be included if he/she had been living full time in the NH for at least 24 hours and was present at 8 AM on the day of the survey. |
| HALT-3 | 24 European countries | 2016-2017 | A resident was eligible to be included if he/she had been living full time in the NH for at least 24 hours and was present at 8 AM on the day of the survey. |
| Heudorf, 2012 | Germany | 2011 | Residents present in the home on the day of the survey. |
| Moro, 2013? | Italy | 2010 | All residents living in the facilities were included, unless absent or hospitalised during the previous 24 hours. |
| Rummukainen, 2009 | Finland | 2005-2008 | Residents present in facilities at 8am on day of survey. |
| Selcuk, 2019 | Singapore | 2008 | Residents were eligible for the PPS if they were residing in the NH at 8:00 a.m., and for at least 24 h, on the day of the survey. |
| Smith, 2013 | Australia | 2011 | Residents were eligible for the PPS if they were residing in the NH at 8:00 a.m., and for at least 24 h, on the day of the survey. |
| Stepan, 2018 | Slovenia | 2016 | All residents were eligible, however only those who were receiving systemic antibiotic treatment and who gave consent to the study were included in the study analysis. |
| Stuart, 2012 | Australia | 2011 | All residents present on day of survey. |
| Szabo, 2014 | Hungary | 2013 | Residents were considered eligible for the survey if (1) she/he lived permanently in the LTCF, (2) had resided there for at least one day and (3) was present at 8 AM on the day of the survey. |
| Thompson, 2016 | US | 2014 | All residents who had been in the facility for >24 hours before the prevalence survey date were eligible for inclusion. |
| Thompson, 2021 | US | 2017 | Residents were considered eligible for the survey if they (1) had resided there for at least one day and (2) were present at 8 AM on the day of the survey. |
| Warren, 1991 | US | 1985 | All residents aged 65 years or older present in the nursing homes on the day of the study. |

PPS is point prevalence survey.
