# Supplementary material for: Temporal and regional trends of antibiotic use in long-term aged care facilities across 39 countries, 1985-2019: Systematic review and meta-analysis
Source: PLoS One. 2021 Aug 23;16(8):e0256501. doi: 10.1371/journal.pone.0256501 (PMC8382177; doi:10.1371/journal.pone.0256501)
Supplement: S6 File — (DOCX) [file pone.0256501.s006.docx]

**S6 File: Detailed heterogeneity statistics**

These tables contain detailed heterogeneity statistics for each meta-analysis estimated from fixed effects models. For our main analysis, the Tau^2^ estimates within each region were pooled and the pooled estimate was applied to generate confidence intervals for each region. Thus, the overall Tau^2^ estimate presented in these tables is not the one used to estimate the confidence intervals, which is presented in the main paper.

Table A: Heterogeneity statistics for point prevalence meta-analysis

| **Region or country** | **Q-statistic** | **I^2^** | **Tau^2^** |
| --- | --- | --- | --- |
| Singapore | 0.841 (1 df, p=0.359) | 0.000 | 0.000 |
| Australia | 111.9 (6 df, p<0.001) | 94.6 | 0.026 |
| British Isles | 291.7 (21 df, p<0.001) | 92.8 | 0.063 |
| Eastern Europe | 592.4 (26 df, p<0.001) | 95.6 | 0.493 |
| North America | 143.9 (3 df, p<0.001) | 97.9 | 0.068 |
| Northern Europe | 1252.4 (23 df, p<0.001) | 98.2 | 0.233 |
| Southern Europe | 696.3 (15 df, p<0.001) | 97.8 | 0.249 |
| Western Europe | 733.0 (20 df, p<0.001) | 97.3 | 0.224 |
| Total within | 3822.3 (115 df, p<0.001) |  |  |
| Total between | 4651.7 (7 df, p<0.001) |  |  |
| Overall | 8473.9 (122 df, p<0.001) | 98.6 | 0.305 |

Table B: Heterogeneity statistics for 12-month period prevalence meta-analysis

| **Region or country** | **Q-statistic (degrees of freedom [df], p-value)** | **I^2^** | **Tau^2^** |
| --- | --- | --- | --- |
| Australia | 1172.1 (6 df, p<0.001) | 99.5 | 0.019 |
| British Isles | 600.0 (5 df, p<0.001) | 99.12 | 0.022 |
| North America | 6532.3 (4 df, p<0.001) | 99.9 | 0.352 |
| Netherlands | n.a* | n.a | n.a |
| Total within | 8304.4 (15 df, p<0.001) |  |  |
| Total between | 28939.3 (3 df, p<0.001) |  |  |
| Overall | 37243.6 (18 df, p<0.001) | 100.0 | 0.210 |

n.a. is ‘not applicable’.

*One study in this group, hence there is no calculation of these statistics.

Table C: Heterogeneity statistics for the percentage of appropriate antibiotic courses as assessed against the McGeer criteria

| **Region or country** | **Q-statistic (degrees of freedom [df], p-value)** | **I^2^** | **Tau^2^** |
| --- | --- | --- | --- |
| Australia | 1478.1 (5 df, p<0.001) | 99.7 | 0.858 |
| England | n.a* | n.a* | n.a* |
| Italy | n.a* | n.a* | n.a* |
| Total within | 1478.1 (5 df, p<0.001) |  |  |
| Total between | 352.0 (2 df, p<0.001) |  |  |
| Overall | 1828.5 (7 df, p<0.001) | 99.6 | 0.920 |

n.a. is ‘not applicable’.

*One study in this group, hence there is no calculation of these statistics.
