# Supplementary material for: Temporal and regional trends of antibiotic use in long-term aged care facilities across 39 countries, 1985-2019: Systematic review and meta-analysis
Source: PLoS One. 2021 Aug 23;16(8):e0256501. doi: 10.1371/journal.pone.0256501 (PMC8382177; doi:10.1371/journal.pone.0256501)
Supplement: S7 File — (DOCX) [file pone.0256501.s007.docx]

**S7 File: Resident characteristics and inclusion criteria reported in studies included in 12-month period prevalence antibiotic use meta-analysis**

Table A. Resident characteristics reported in studies included in 12-month period prevalence antibiotic use meta-analysis

| **Region / Study name** | **Country** | **Year** | **Mean age** | **Median age** | **% >85 years** | **% female** | **% with urinary catheter** | **% with indwelling medical devices** | **% with dementia** | **% with cognitive impairment** | **% with wounds (other than pressure sores)** | **% with pressure sores** |
| --- | --- | --- | --- | --- | --- | --- | --- | --- | --- | --- | --- | --- |
| **Australia** | | | | | | | | | | | | |
| Taxis, 2017 | Australia | 2009 | 85.8 | - | - | 70.3 | - | - | - | - | - | - |
| Cowan, 2016 | Australia | 2014 | - | - | - | 62.0 | - | - | - | - | - | - |
| Raban, 2020 | Australia | 2015 | 85.8 | - | - | 69.0 | - | 3.3 | 56.7 | - | 5.7 | - |
|  |  | 2016 | 85.8 | - | - | 67.8 | - | 3.2 | 55.1 | - | 5.4 | - |
| Sluggett, 2020 | Australia | 2005 | - | - | - | 68.4 | - | - | - | - | - | - |
|  |  | 2010 | - | - | - | 69.7 | - | - | - | - | - | - |
|  |  | 2015 | - | - | - | 68.2 | - | - | - | - | - | - |
| **British Isles** | | | | | | | | | | | | |
| Gillespie,2015 | Wales | 2011 |  | 86 | 79.6* | 75.9 | - | - | - | - | - | - |
| Sundvall, 2015 | UK | 2011 | 87 | - | - | 73 | 4.4 | - | - | - | - | - |
| Thornley, 2019 | England | 2017 | - | - | - | - | - | - | - | - | - | - |
| Thornley, 2019 | Scotland | 2017 | - | - | - | - | - | - | - | - | - | - |
| Thornley, 2019 | Wales | 2017 | - | - | - | - | - | - | - | - | - | - |
| Thornley, 2019 | Ireland | 2017 | - | - | - | - | - | - | - | - | - | - |
| **North America** | | | | | | | | | | | | |
| Warren, 1991 | US | 1985 | - | - | - | - | - | - | - | - | - | - |
| Montgomery, 1995 | Canada | 1986 | 85 | - | - | 72 | 5.5 | - | - | - | - | - |
| Myolette, 1996 | US | 1989 | - | - | - | - | 12† | - | - | - | - | - |
| Daneman, 2013 | Canada | 2010 | - | 86 | - | 72 | 4.2 | - | 56.7 | - | - | - |
| Daneman, 2017 | Canada | 2014 | - | - | - | - | 4‡ | - | 65‡ | - | - | - |
| **Northern Europe** | | | | | | | | | | | | |
| Taxis, 2017 | Netherlands | 2009 | 82.8 | - | - | 68.2 | - | - | - | - | - | - |

*Percentage of residents aged ≥ 80 years.

†Average percentage of catheter days per month of total resident days.

‡Median prevalence across facilities.

Table B. Residents eligible for inclusion in studies included in 12-month period prevalence antibiotic use meta-analysis

| **Study** | **Eligibility criteria** |
| --- | --- |
| Cowan, 2016 | Not clear. 178 included from 216. |
| Daneman, 2013 | All residents aged ≥ 66 years. |
| Daneman, 2017 | All residents of doctors who prescribed at least 100 LTCF prescriptions over 2 years. |
| Gillespie,2015 | Residents were eligible if they had been admitted to the home for >24 h with a planned admission for longer than one month. There were no exclusion criteria. |
| Montgomery, 1995 | 20% probability sample of all residents. |
| Myolette, 1996 | All residents. |
| Raban, 2020 | Residents were eligible if they were: 1) permanent residents (i.e. not users of respite care), 2) aged ≥ 65 years; and 3) at the facility for a minimum length of stay of two weeks |
| Sluggett, 2020 | Residents were eligible if they were: 1) non-Aboriginal individuals, 2) aged ≥ 65 years; 3) long-term residents (individuals who received residential aged care services for a minimum of 100 consecutive days) |
| Sundvall, 2015 | Residents aged ≥ 75 years residing in care homes. |
| Taxis, 2017 | All residents present in facilities. |
| Thornley, 2019a | All residents. |
| Warren, 1991 | All residents aged ≥ 65 years residing in care home on the first day of the study. |

LTCF is long-term care facility.
