# Supplementary material for: Temporal and regional trends of antibiotic use in long-term aged care facilities across 39 countries, 1985-2019: Systematic review and meta-analysis
Source: PLoS One. 2021 Aug 23;16(8):e0256501. doi: 10.1371/journal.pone.0256501 (PMC8382177; doi:10.1371/journal.pone.0256501)
Supplement: S8 File — (DOCX) [file pone.0256501.s008.docx]

**S8 File: Antibiotic use in long-term aged care facilities (studies not included in meta-analyses)**

Table A. Percentage of residents on an antibiotic

| **Author, year** | **Country** | **Number of facilities** | **Data collection year/s** | **Period of data collection** | **Period prevalence [median (IQR)]** | **Period prevalence (%)** | **Mean point prevalence**  **(95% CI)** |
| --- | --- | --- | --- | --- | --- | --- | --- |
| Barney, 2019(1) | United States | 4 | 2016 - 2017 | 6 months | 6.7 (5.0-8.6) |  |  |
| Benoit, 2008(2) | United States | 73 | 2001 – 2002 | 6 months |  | 42.0 |  |
| Eikelenboom-Boskamp, 2019(3) | Netherlands | 25 up to 44^a^ | 2010 – 2017 | 1 month |  |  | 6.0 (5.5 – 7.0) |
| Eriksen, 2013(4) | Norway | 5 | 2009 | 1 month  (April then November) |  | 8.6 (Apr); 8.1 (Nov) |  |
| Lee, 1992(5) | United States | 7 | 1989 | 3-4 months^b^ |  | 33.0 |  |
| Mayne, 2018(6) | Australia | 5 | 2015 – 2016 | 1 month |  | 20.0 |  |
| Monette, 2007(7) | Canada | 4  (intervention) | 2001 – 2003 | 3 months |  | 20.0 |  |
|  |  | 4  (control) | 2001 - 2003 | 3 months |  | 13.0 |  |
| Pakyz, 2010(8) | United States | 1174 | 2004 | 6 months |  | 10.8 |  |
| Selcuk, 2018(9) | Singapore | 4 | 2008 | 1 month |  | 2.33 |  |

^a^: Point prevalence was conducted each year from 2010 through to 2017, with the mean prevalence reported. The number of participating LTCFs increase from 25 in 2010 up to 44 in 2017.

^b^: Four facilities were studied for 4 months (April—July 1989) and three for 3 months (either April-June 1989 or July-September 1989).

Table B. Antibiotic use in long-term aged care facilities reported as days of therapy (DOT) per 1000 resident days, courses per 1000 resident days, and defined daily doses (DDD) per 1000 resident days

| **Author, year** | **Country** | **Number of facilities** | **Data collection year/s** | **DOT/1000 Resident Days**  **Mean (SD)** | **Course/1000 Resident Days**  **Mean (SD)** | **DDD/1000 Resident Days**  **Mean (SD)** | **Bed Days** |
| --- | --- | --- | --- | --- | --- | --- | --- |
| Barney, 2019(1) | United States | 4 | 2016 - 2017 | 68.9 (median) 54.4–100.6 (IQR) |  |  | NR |
| Benoit, 2008(2) | United States | 73 | 2001 - 2002 |  | 4.8 (0.4-23.5) |  | NR |
| Blix, 2007(10) | Norway | 133 | 2003 |  |  | 148 (70.1) | NR |
| Daneman, 2015(11) | Canada | 607 | 2010-2011 | 55.0 (20.4) |  |  | 50,953,000 |
| Fagan, 2012(12) | Norway | 10 | 2007 – 2008 |  |  | 55 | 111,091 |
| Felsen, 2020(13) | United States | 6 | 2014 | 80.5 |  |  | NR |
| Fleet, 2014(14) | England | 15 (intervention) | 2010 |  |  | 69.8 (range: 31.8-122.4), | NR |
|  |  | 15 (control) | 2010 |  |  | 49.7 (range: 17.6-122.1) | NR |
| Gillespie, 2015(15) | Wales | 10 | 2010 - 2012 |  | 2.1 (range: 0.9-2.9) |  | 310 (median BD/resident) |
| Ishikane, 2020(16) | Japan | 6 | 2016 |  |  | 15.3 (range: 3.0 – 69.2) | NR |
| Jump, 2012(17) | United States | 1 | 2006 - 2010 | 175.1 (28.0) |  |  | NR |
| Kabbani, 2019 | United States | 12 | 2016 | 54.0 (25.0-133.0 IQR) |  |  | NR |
| Katz, 1990(18) | United States | 2 | 1985 |  | 12.1 |  | 18000 |
| Loeb, 2005(19) | United States | 12 (intervention) | 2001-2002 |  | 3.52 |  | 2337 (courses) |
|  | United States | 12 (control) |  |  | 3.93 |  | 2569 (courses) |
| Marquet, 2015(20) | France | 52 | 2011 |  |  | 39.0 | NR |
|  |  | 52 | 2012 |  |  | 39.3 | NR |
|  |  | 74 | 2013 |  |  | 44.8 | NR |
| Marra, 2017(21) | Canada | 381 | 2007 |  | 5.8 (mean from 2007 – 2014) | 39.2 | NR |
|  |  | 381 | 2014 |  |  | 35.2 | NR |
| Mylotte, 1999(22) | United States | 4 | 1996-1998 | 47.5 (6.75) |  |  | NR |
|  |  | 1 |  |  | 6.3 (2.5) |  |  |
|  |  | 1 |  |  | 4.1 (2.0) |  |  |
|  |  | 1 |  |  | 4.0 (1.4) |  |  |
|  |  | 1 |  |  | 7.2 (2.0) |  |  |
| Mylotte, 2005(23) | United States | 11 | 2003 | 115.6 (36.5) |  |  | NR |
| Natsch, 1998(24) | Netherlands | 6 | 1995 |  |  | 64.7 | NR |
| Olsho, 2013(25) | United States | 12 | 2011 |  | 11.5 (range: 5.4 – 25.9) |  | 11,810 |
| Pluss-Suard, 2019(26) | Switzerland | 23 | 2011 |  |  | 45.6 (range: 5.9-103.9) | NR |
| Raban, 2020(27) | Australia | 68 | 2015 | 81.57 (6.19) | 7.60 (0.41) |  | 5,230,215 (for 2015-17) |
|  |  |  | 2016 | 83.3 (5.34) | 7.81 (0.31) |  |  |
|  |  |  | 2017* | 85.06 (6.51) | 8.01 (0.45) |  |  |
| Rahme, 2016(28) | United States | 1 | 2012-2013 |  |  | 82.3 (12.2) | NR |
| Roukens, 2017(29) | Netherlands | 31 | 2012-2014 |  |  | 73.5 | 25,184 |
| Selcuk, 2018(9) | Singapore | 4 | 2008 | 24.8 |  | 28.9 | NR |
| Selcuk, 2019(30) | Singapore | 9 | 2017 |  |  | 38.2 (oral only) | NR |
| Sloane, 2014(31) | United States | 4 | 2010 – 2012 |  | 3.4 |  | 59,883 |
| Sloane, 2019(32) | United States | 14 | 2015-2017 |  | 11.9 |  | NR |
| Sloane, 2020(33) | United States | 14 | NR | 11.92  (intervention) |  |  | NR |
|  | United States | 13 | NR | 12.91  (control) |  |  | NR |
| Sluggett, 2020(34) | Australia | 3218 | 2005 |  |  | 67.6 | 22,228,297 |
|  |  |  | 2010 |  |  | 74.6 | 40,637,942 |
|  |  |  | 2015 |  |  | 93.8 | 48,861,838 |
| Smith, 2020(35) | United Kingdom | 135 | 2016 |  | 2.68 prescriptions per resident year (95% confidence interval (CI) 2.64–2.71) |  | 3,916,931 |
| Song, 2021(36) | United States | 29 | 2016 | 237 (144.4) | 37.6 (23.2) |  | NR |
| Stuart, 2015(37) | Australia | 2 | 2012 | 723 (62) |  |  | 11,661 |
| Takito, 2020(38) | Japan | 1 | 2013 |  | 14.10 prescriptions per 100 residents per month |  | NR |
|  |  | 1 | 2014 |  | 18.51 prescriptions per 100 residents per month |  | NR |
| Temime, 2018(39) | France | 13 | 2014 (intervention) |  |  | 54 | NR |
|  |  | 13 | 2014 (control) |  |  | 52 | NR |
| van Buul, 2015(40) | Netherlands | 5 | 2012 – 2013 (intervention) | 5.5 |  | 62.3 | NR |
|  |  | 5 | 2012 – 2013  (control) | 4.6 |  | 46.2 | NR |
| Warren, 1991(41) | United States | 52 | 1985 - 1986 |  | 4.6 |  | 1,148,161 |
| Wu, 2015(42) | Canada | 17 | 2011 - 2012 | 2.7 (IQR: 2.1-3.0) |  |  | 162,080 |
| Zimmerman, 2014(43) | United States | 12 | 2011 |  | 13.0 |  | 336,522 |

NR is not reported.

*Based on data from Jan-Sep 2017.

Table C: Studies assessing appropriateness of antibiotic therapy by criteria other than the McGeer criteria

| **Author, Year** | **Country** | **Data collection year** | **Data collection period** | **Specific indication?** | **% of appropriate prescriptions** | **No. of appropriate prescriptions** | **No. of prescriptions or courses assessed** | **Denominator type** | **Basis of appropriateness** |
| --- | --- | --- | --- | --- | --- | --- | --- | --- | --- |
| **All infections** | | | | | | | | | |
| Fleet, 2014(14) | England | 2010 - 2011 | January – May, 2010 & January - May, 2011 | Any infection | 11.5 | 94.9 | 825 | Courses | Loeb Minimum Criteria |
| Fleet, 2014(14) | England | 2010 - 2011 | January – May, 2010 & January - May, 2011 | Any infection | 12.6 | 101.1 | 803 | Courses | Loeb Minimum Criteria |
| Olsho, 2013(25) | US | 2011 | March - May | Any infection | 12.7 | 429.4 | 3381 | Prescriptions | Loeb Minimum Criteria |
| Smith, 2013(44) | Australia | 2011 | July | Any infection | 97 | 65.0 | 67 | Prescriptions | Australian consensus guidelines for antimicrobial therapy |
| McClean, 2011a(45) | Northern Ireland | 2009 | April | Any infection | 81.9 | 794.4 | 970 | Prescriptions | British National Foundry (version 61) and the Northern Ireland Antimicrobial Guidelines for Primary Care |
| McClean, 2011a(45) | Northern Ireland | 2009 | November | Any infection | 80.4 | 791.1 | 984 | Prescriptions | British National Foundry (version 61) and the Northern Ireland Antimicrobial Guidelines for Primary Care |
| McClean, 2012(46) | Northern Ireland | 2010 | November | Any infection | 76.8 | 652.8 | 850 | Prescriptions | British National Foundry (version 62) and the Northern Ireland Antimicrobial Guidelines for Primary Care |
| McClean, 2012(46) | Northern Ireland | 2011 | April | Any infection | 72 | 395.3 | 549 | Prescriptions | British National Foundry (version 62) and the Northern Ireland Antimicrobial Guidelines for Primary Care |
| Sloane, 2014(31) | US | 2010 - 2011 | August, 2010 - February, 2011 | Any infection | 60 | 18 | 30 | Prescriptions | Expert panel determined to be "Definitely appropriate" |
| Moro, 2013(47) | Italy | 2010 | May - September | Any infection | 78 | 95.2 | 122 | Courses | Infection was suspected or diagnosed |
| Boivin, 2013(48) | France | 2012 | April - July | Any infection | 88.5 | 46.9 | 53 | Prescriptions | Infectious Diseases Society of America Guidelines (2008) |
| van Buul, 2015(40) | Netherlands | 2012 | January - September | Any infection | 79 | 219.6 | 278 | Prescriptions | In-house algorithm |
| van Buul, 2015(40) | Netherlands | 2012 | January - September | Any infection | 70 | 224 | 320 | Prescriptions | In-house algorithm |
| Monette, 2007(7) | Canada | 2001 – 2003 | December, 2001 – February, 2003 | Any infection | 59.2 | 92.9 | 157 | Prescriptions | In-house Guidelines |
| Monette, 2007(7) | Canada | 2001 – 2003 | December, 2001 – February, 2003 | Any infection | 56.2 | 154.0 | 274 | Prescriptions | In-house Guidelines |
| Cowan, 2016(49) | Australia | 2014 | "278 chart days" | Any infection | 19.5 | 96.7 | 496 | Prescriptions | Microbiology lab results |
| Fleet, 2014(14) | England | 2010 - 2011 | January – May, 2010 & January - May, 2011 | Any infection | 5.6 | 46.2 | 825 | Courses | Microbiology lab results |
| Fleet, 2014(14) | England | 2010 - 2011 | January – May, 2010 & January - May, 2011 | Any infection | 8.7 | 69.9 | 803 | Courses | Microbiology lab results |
| Alberg, 2017(50) | Norway | 2016 | May | Any infection | Range: 0-60 | NR | 966 | Courses | Microbiology lab results |
| Alberg, 2017(50) | Norway | 2016 | May | Any infection | Range: 0-77 | NR | 966 | Courses | Norwegian National Guidelines (2000) |
| Fagan, 2012(12) | Norway | 2007 - 2008 | March, 2007 - February, 2008 | Any infection | 77.3 | 481.6 | 623 | Courses | Norwegian National Guidelines (2000) |
| Wu, 2015(42) | Canada | 2011 - 2012 | April, 2011 to March, 2012 | Any infection | 23 | 192.1 | 835 | Prescriptions | Positive culture results |
| **Respiratory Tract Infection (RTI)** | | | | | | | | | |
| Fagan, 2012(12) | Norway | 2007 - 2008 | March, 2007 - February, 2008 | RTI | 79 | 116.9 | 148 | Courses | Norwegian National Guidelines (2000) |
| **Skin and Soft Tissue Infections (SSTI)** | | | | | | | | | |
| Fagan, 2012(12) | Norway | 2007 - 2008 | March, 2007 - February, 2008 | SSTI | 76 | 73.7 | 97 | Courses | Norwegian National Guidelines (2000) |
| **Urinary Tract Infections (UTI)** | | | | | | | | | |
| Eure, 2017(51) | US | 2013-2014 | December - May | UTI | 30 | 9.9 | 33 | Residents | Loeb Minimum Criteria |
| Eure, 2017(51) | US | 2013-2014 | December - May | UTI | 45 | 14.9 | 33 | Residents | Crnich algorithm |
| Fagan, 2012(12) | Norway | 2007 - 2008 | March, 2007 - February, 2008 | UTI | 77 | 291.1 | 378 | Courses | Norwegian National Guidelines (2000) |
| McClean, 2011(45) | Northern Ireland | 2009 | April | UTI | 15.4 | 56.5 | 367 | Residents | Urinalysis |
| McClean, 2011(45) | Northern Ireland | 2009 | November | UTI | 27.8 | 127.9 | 460 | Residents | Urinalysis |

**References**

1. Barney GR, Felsen CB, Dumyati GK. One-day point prevalence as a method for estimating antibiotic use in nursing homes. Infection Control and Hospital Epidemiology. 2019;40(2):221-3.

2. Benoit SR, Nsa W, Richards CL, Bratzler DW, Shefer AM, Steele LM, et al. Factors associated with antimicrobial use in nursing homes: a multilevel model. Journal of the American Geriatrics Society. 2008;56(11):2039-44.

3. Eikelenboom-Boskamp A, Saris K, van Loosbroek M, Drabbe MIJ, de Jongh F, de Jong JWD, et al. Prevalence of healthcare-associated infections in Dutch nursing homes: follow-up 2010-2017. The Journal of hospital infection. 2019;101(1):49-52.

4. Eriksen HM, Saether AR, Viktil KK, Andberg L, Munkerud MW, Willoch K, et al. Use of antibiotics in nursing homes--surveillance with different methods. Tidsskrift for Den Norske Laegeforening. 2013;133(19):2052-6.

5. Lee YL, Thrupp LD, Friis RH, Fine M, Maleki P, Cesario TC. Nosocomial infection and antibiotic utilization in geriatric patients: a pilot prospective surveillance program in skilled nursing facilities. Gerontology. 1992;38(4):223-32.

6. Mayne S, Sundvall PD, Gunnarsson R. Confusion Strongly Associated with Antibiotic Prescribing Due to Suspected Urinary Tract Infections in Nursing Homes. Journal of the American Geriatrics Society. 2018;66(2):274-81.

7. Monette J, Miller MA, Monette M, Laurier C, Boivin JF, Sourial N, et al. Effect of an educational intervention on optimizing antibiotic prescribing in long-term care facilities. Journal of the American Geriatrics Society. 2007;55(8):1231-5.

8. Pakyz AL, Dwyer LL. Prevalence of antimicrobial use among United States nursing home residents: results from a national survey. Infection Control & Hospital Epidemiology. 2010;31(6):661-2.

9. Selcuk A, Teng CB, Chan SY, Yap KZ. Antimicrobial use and drug-drug interactions among nursing home residents in Singapore: a multicentre prevalence study. International Journal of Clinical Pharmacy. 2018.

10. Blix HS, Roed J, Sti MO. Large variation in antibacterial use among Norwegian nursing homes. Scandinavian Journal of Infectious Diseases. 2007;39(6-7):536-41.

11. Daneman N, Bronskill SE, Gruneir A, Newman AM, Fischer HD, Rochon PA, et al. Variability in Antibiotic Use Across Nursing Homes and the Risk of Antibiotic-Related Adverse Outcomes for Individual Residents. JAMA Internal Medicine. 2015;175(8):1331-9.

12. Fagan M, Maehlen M, Lindbaek M, Berild D. Antibiotic prescribing in nursing homes in an area with low prevalence of antibiotic resistance: compliance with national guidelines. Scandinavian Journal of Primary Health Care. 2012;30(1):10-5.

13. Felsen CB, Dodds Ashley ES, Barney GR, Nelson DL, Nicholas JA, Yang H, et al. Reducing Fluoroquinolone Use and Clostridioides difficile Infections in Community Nursing Homes Through Hospital-Nursing Home Collaboration. J Am Med Dir Assoc. 2020;21(1):55-61.e2.

14. Fleet E, Gopal Rao G, Patel B, Cookson B, Charlett A, Bowman C, et al. Impact of implementation of a novel antimicrobial stewardship tool on antibiotic use in nursing homes: a prospective cluster randomized control pilot study. Journal of Antimicrobial Chemotherapy. 2014;69(8):2265-73.

15. Gillespie D, Hood K, Bayer A, Carter B, Duncan D, Espinasse A, et al. Antibiotic prescribing and associated diarrhoea: a prospective cohort study of care home residents. Age & Ageing. 2015;44(5):853-60.

16. Ishikane M, Kusama Y, Tanaka C, Hayakawa K, Kuwahara T, Ohmagari N. Epidemiology of Antimicrobial Use among Nursing Homes in Japan, 2016: a Pilot Study. Jpn J Infect Dis. 2020;73(4):293-5.

17. Jump RL, Olds DM, Seifi N, Kypriotakis G, Jury LA, Peron EP, et al. Effective antimicrobial stewardship in a long-term care facility through an infectious disease consultation service: keeping a LID on antibiotic use. Infection Control & Hospital Epidemiology. 2012;33(12):1185-92.

18. Katz PR, Beam TR, Jr., Brand F, Boyce K. Antibiotic use in the nursing home. Physician practice patterns. Archives of Internal Medicine. 1990;150(7):1465-8.

19. Loeb M, Brazil K, Lohfeld L, McGeer A, Simor A, Stevenson K, et al. Effect of a multifaceted intervention on number of antimicrobial prescriptions for suspected urinary tract infections in residents of nursing homes: cluster randomised controlled trial. BMJ. 2005;331(7518):669.

20. Marquet A, Thibaut S, LePabic E, Huon JF, Ballereau F. Three years of antibiotic consumption evaluation in French nursing homes. Medecine et Maladies Infectieuses. 2015;45(8):313-7.

21. Marra F, McCabe M, Sharma P, Zhao B, Mill C, Leung V, et al. Utilization of Antibiotics in Long-Term Care Facilities in British Columbia, Canada. Journal of the American Medical Directors Association. 2017;18(12):1098.e1-.e11.

22. Mylotte JM. Antimicrobial prescribing in long-term care facilities: prospective evaluation of potential antimicrobial use and cost indicators. American Journal of Infection Control. 1999;27(1):10-9.

23. Mylotte JM, Keagle J. Benchmarks for antibiotic use and cost in long-term care. Journal of the American Geriatrics Society. 2005;53(7):1117-22.

24. Natsch S, Hekster YA, de Jong R, Heerdink ER, Herings RM, van der Meer JW. Application of the ATC/DDD methodology to monitor antibiotic drug use. European Journal of Clinical Microbiology & Infectious Diseases. 1998;17(1):20-4.

25. Olsho LE, Bertrand RM, Edwards AS, Hadden LS, Morefield GB, Hurd D, et al. Does adherence to the Loeb minimum criteria reduce antibiotic prescribing rates in nursing homes? Journal of the American Medical Directors Association. 2013;14(4):309.e1-7.

26. Pluss-Suard C, Niquille A, Hequet D, Krahenbuhl S, Pichon R, Zanetti G, et al. Decrease in Antibacterial Use and Facility-Level Variability After the Introduction of Guidelines and Implementation of Physician-Pharmacist-Nurse Quality Circles in Swiss Long-term Care Facilities. Journal of the American Medical Directors Association. 2020;21(1):78-83.

27. Raban MZ, Lind KE, Day RO, Gray L, Georgiou A, Westbrook JI. Trends, determinants and differences in antibiotic use in 68 residential aged care homes in Australia, 2014-2017: a longitudinal analysis of electronic health record data. BMC Health Serv Res. 2020;20(1):883.

28. Rahme CL, Jacoby HM, Avery LM. Impact of a Hospital’s Antibiotic Stewardship Team on Fluoroquinolone Use at a Long-Term Care Facility. Annals of Long-Term Care: Clinical Care and Aging. 2016;24(6):13-20.

29. Roukens M, Verhoef L, Stobberingh E, Natsch S. Surveillance of antimicrobial use in Dutch long-term care facilities. Journal of Antimicrobial Chemotherapy. 2017;72(5):1516-20.

30. Selcuk A, Yap KZ, Wong CL, Yang JX, Yong PC, Chan SY, et al. A Point Prevalence Study of Antimicrobial Use and Practice Among Nursing Homes in Singapore. Drugs Aging. 2019;36(6):559-70.

31. Sloane PD, Zimmerman S, Reed D, Beeber AS, Chisholm L, Kistler C, et al. Antibiotic prescribing in 4 assisted-living communities: incidence and potential for improvement. Infection Control & Hospital Epidemiology. 2014;35 Suppl 3:S62-8.

32. Sloane PD, Zimmerman S, Ward K, Kistler CE, Paone D, Weber DJ, et al. A 2-Year Pragmatic Trial of Antibiotic Stewardship in 27 Community Nursing Homes. Journal of the American Geriatrics Society. 2019.

33. Sloane PD, Zimmerman S, Ward K, Kistler CE, Paone D, Weber DJ, et al. A 2-Year Pragmatic Trial of Antibiotic Stewardship in 27 Community Nursing Homes. Journal of the American Geriatrics Society. 2020.

34. Sluggett JK, Moldovan M, Lynn DJ, Papanicolas LE, Crotty M, Whitehead C, et al. National Trends in Antibiotic Use in Australian Residential Aged Care Facilities, 2005-2016. Clin Infect Dis. 2020.

35. Smith CM, Williams H, Jhass A, Patel S, Crayton E, Lorencatto F, et al. Antibiotic prescribing in UK care homes 2016-2017: retrospective cohort study of linked data. BMC Health Serv Res. 2020;20(1):555.

36. Song S, Wilson BM, Bej T, Gravenstein S, Carter RR, Marek J, et al. Antibiotic Use Among Residents Receiving Skilled Nursing Care in 29 U.S. Nursing Homes. Journal of the American Geriatrics Society. 2021;69(2):399-406.

37. Stuart RL, Orr E, Kotsanas D, Gillespie EE. A nurse-led antimicrobial stewardship intervention in two residential aged care facilities. Healthcare Infection. 2015;20(1):4-6.

38. Takito S, Kusama Y, Fukuda H, Kutsuna S. Pharmacist-supported antimicrobial stewardship in a retirement home. J Infect Chemother. 2020;26(8):858-61.

39. Temime L, Cohen N, Ait-Bouziad K, Denormandie P, Dab W, Hocine MN. Impact of a multicomponent hand hygiene-related intervention on the infectious risk in nursing homes: A cluster randomized trial. Am J Infect Control. 2018;46(2):173-9.

40. van Buul LW, van der Steen JT, Achterberg WP, Schellevis FG, Essink RT, de Greeff SC, et al. Effect of tailored antibiotic stewardship programmes on the appropriateness of antibiotic prescribing in nursing homes. Journal of Antimicrobial Chemotherapy. 2015;70(7):2153-62.

41. Warren JW, Palumbo FB, Fitterman L, Speedie SM. Incidence and characteristics of antibiotic use in aged nursing home patients. Journal of the American Geriatrics Society. 1991;39(10):963-72.

42. Wu LDY, Walker SAN, Elligsen M, Paimay L, Simor A, Daneman N. Antibiotic use and need for antimicrobial stewardship in long-term care. Canadian Journal of Hospital Pharmacy. 2015;68(6):445-9.

43. Zimmerman S, Sloane PD, Bertrand R, Olsho LE, Beeber A, Kistler C, et al. Successfully reducing antibiotic prescribing in nursing homes. Journal of the American Geriatrics Society. 2014;62(5):907-12.

44. Smith M, Atkins S, Worth L, Richards M, Bennett N. Infections and antimicrobial use in Australian residential aged care facilities: a comparison between local and international prevalence and practices. Australian Health Review. 2013;37(4):529-34.

45. McClean P, Tunney M, Gilpin D, Parsons C, Hughes C. Antimicrobial prescribing in nursing homes in Northern Ireland: results of two point-prevalence surveys. Drugs & aging. 2011;28(10):819-29.

46. McClean P, Tunney M, Gilpin D, Parsons C, Hughes C. Antimicrobial prescribing in residential homes. Journal of Antimicrobial Chemotherapy. 2012;67(7):1781-90.

47. Moro ML, Ricchizzi E, Morsillo F, Marchi M, Puro V, Zotti CM, et al. Infections and antimicrobial resistance in long term care facilities: a national prevalence study. Annali di Igiene. 2013;25(2):109-18.

48. Boivin Y, Talon D, Leroy J, Floret N, Gbaguidi-Haore H, Bertrand X. Antibiotic prescription in nursing homes for dependent elderly people: a cross-sectional study in Franche-Comte. Medecine et Maladies Infectieuses. 2013;43(4):163-9.

49. Cowan RU, Kishan D, Walton AL, Sneath E, Cheah T, Butwilowsky J, et al. Cleaning, resistant bacteria, and antibiotic prescribing in residential aged care facilities. American Journal of Infection Control. 2016;44(3):e19-21.

50. Alberg T, Holen O, Blix HS, Lindbaek M, Bentele H, Eriksen HM. Antibiotic use and infections in nursing homes. Tidsskrift for Den Norske Laegeforening. 2017;137(5):357-61.

51. Eure T, LaPlace LL, Melchreit R, Maloney M, Lynfield R, Whitten T, et al. Measuring Antibiotic Appropriateness for Urinary Tract Infections in Nursing Home Residents. Infection Control & Hospital Epidemiology. 2017;38(8):998-1001.
