# Supplementary material for: Temporal and regional trends of antibiotic use in long-term aged care facilities across 39 countries, 1985-2019: Systematic review and meta-analysis
Source: PLoS One. 2021 Aug 23;16(8):e0256501. doi: 10.1371/journal.pone.0256501 (PMC8382177; doi:10.1371/journal.pone.0256501)
Supplement: S9 File — (DOCX) [file pone.0256501.s009.docx]

**S9 File:** **Resident characteristics reported in studies included in appropriateness of antibiotic use (according to McGeer criteria) meta-analysis**

| **Country / Study name** | **Year** | **Mean age** | **Median age** | **% >85 years** | **% female** | **% with urinary catheter** | **% with indwelling medical devices** | **% with dementia** | **% with cognitive impairment or disorientated** | **% with wounds (other than pressure sores)** | **% with pressure sores** |
| --- | --- | --- | --- | --- | --- | --- | --- | --- | --- | --- | --- |
| **Australia** | | | | | | | | | | | |
| acNAPS, 2016 | 2015 | - | - | 47.7 | 65.6 | 4.3 | - | - | - | - | - |
| acNAPS, 2017 | 2016 | - | - | 54.3 | 67.1 | 3.8 | - | - | - | - | - |
| acNAPS, 2018 | 2017 | - | - | 57.9 | 66.0 | 3.8 | - | - | - | - | - |
| acNAPS, 2020 | 2018 | - | - | 59.4 | 67.3 | 3.8 | - | - | - | - | - |
| Stuart, 2012 | 2011 | - | - | 28 | 51 | 3 | - | - | 80 | - | 5 |
| Stuart, 2015 | 2012 | - | - | - | - | - | - | - | - | - | - |
| **England** | | | | | | | | | | | |
| Fleet, 2014 | 2010 | - | - | IG: 46.7  CG: 44.1 | IG: 65.9  CG: 67.6 | - | IG: 11.2  CG: 6.6 | - | - | IG: 10.6  CG: 9.3 | - |
| **Italy** | | | | | | | | | | | |
| Moro, 2013 (HALT-1) | 2010 | - | - | 48.7 | 76.3 | 12.3 | - | - | 64.2 | 7.3 | 8.8 |
| **US** | | | | | | | | | | | |
| Eure, 2017 |  | - | - | - | - | - | - | - | - | - | - |
