# Supplementary material for: Temporal and regional trends of antibiotic use in long-term aged care facilities across 39 countries, 1985-2019: Systematic review and meta-analysis
Source: PLoS One. 2021 Aug 23;16(8):e0256501. doi: 10.1371/journal.pone.0256501 (PMC8382177; doi:10.1371/journal.pone.0256501)
Supplement: S10 File — (DOCX) [file pone.0256501.s010.docx]

**S10 File: Number of studies measuring antibiotic use in LTCF included in this systematic review by year**
