# Supplementary material for: Temporal and regional trends of antibiotic use in long-term aged care facilities across 39 countries, 1985-2019: Systematic review and meta-analysis
Source: PLoS One. 2021 Aug 23;16(8):e0256501. doi: 10.1371/journal.pone.0256501 (PMC8382177; doi:10.1371/journal.pone.0256501)
Supplement: Attachment — Submitted filename: Response to reviewers_Raban et al2.docx [file pone.0256501.s011.docx]

**RESPONSE TO REVIEWERS**

Title:	Temporal and regional trends of antibiotic use in long-term aged care facilities across 39 countries, 1985-2019: systematic review and meta-analysis

Authors:	Raban MZ, Gates PJ, Gasparini C, Westbrook JI

Dear Prof Karunasagar,

We are pleased to submit a revision of our article to PLoS One after addressing reviewer comments. We would like to thank the Reviewers and Editors for their careful review of our manuscript. Below is our detailed response to the comments.

Sincerely yours,

Magdalena Raban

**Editor’s comments**

|  | **Comment** | **Response** |
| --- | --- | --- |
| 7 | Please see the reviewer comments. There seems to be some discrepancy in the stated years of study and the data included in the study. The authors also need to address the question on macrolide and sulphonamides. Please address al reviewer questions point by point. | We have addressed the reviewer comments point by point. |
| 8 | We note that this manuscript is a systematic review or meta-analysis; our author guidelines therefore require that you use PRISMA guidance to help improve reporting quality of this type of study. Please upload copies of the completed PRISMA checklist as Supporting Information with a file name “PRISMA checklist”. | We have completed the PRISMA checklist and this is uploaded with the filename “PRISMA_checklist”. Furthermore, it is referred to as “Supplementary file S2” in the manuscript. |
| 9 | Please review your reference list to ensure that it is complete and correct. If you have cited papers that have been retracted, please include the rationale for doing so in the manuscript text, or remove these references and replace them with relevant current references. Any changes to the reference list should be mentioned in the rebuttal letter that accompanies your revised manuscript. If you need to cite a retracted article, indicate the article’s retracted status in the References list and also include a citation and full reference for the retraction notice. | We have reviewed our reference list. We have not included any retracted articles, nor have we added or removed citations in this revision. We have tidied up the journal names to ensure they all comply with PLoS One’s referencing guideline. |

**Reviewer 1**

|  | **Comment** | **Response** |
| --- | --- | --- |
| 1 | The review seems more of Europe representation. The other areas have statistically less representation as one two studies from Singapore or 1 from Australia. This could bring about bias | We acknowledge the origin of the data and have included country labels for each data point so this is transparent to the reader. Furthermore, we have provided estimates by region to account for region differences, including availability of data. |
| 2 | The said period was 1990 to 2021. Studies from 1985 are shown to be included. | The search strategy was run to identify studies published from 1990. However, these studies could include data prior to 1990. For example, in Table 1, the study “Katz, 1990” was published in 1990 but reported data from 1985. Similarly, “Lee 1992” reported data from 1989; and the most recent studies published in 2020 only reported data up to 2019.  To make this clearer, we have added the following in “Methods, Information sources and search strategy” (new text in italics):  “We searched the bibliographic databases Medline (via OvidSP and PubMed) and EMBASE (via OvidSP) for original research articles reporting rates of antibiotic use in LTCFs published in English between 1990-2021. *Studies could report data prior to 1990*.” (p. 6)  We have also added the following in “Methods, Data collection process and data items”:  “For each study data on: the study country, sample size (number of LTCFs and residents), study design (point prevalence survey, longitudinal cohort study, intervention study), period of data collection (*which could precede 1990)*…”. (p. 7) |
| 3 | Need to look into the data of Macrolides and sulphonamides use. These are widely used in the community setup for respiratory or urinary tract infections. | We agree with the reviewer that macrolides and sulphonamides are commonly used in LTCFs, particularly since respiratory and urinary tract infections are the most common infection types, along with skin and soft tissue infections. In Figure 4 we present the classes of antibiotics reported to be among the three most frequently used in each study. We refer to the numbers of studies reporting these classes in the three most frequently used classes. Macrolides were only reported as the most frequent class used in four of 59 studies. Examining the use of these classes in more detail was beyond the scope of this study. |
| 4 | It would be interesting to note if the quinolones usage was before 2015 or it remains so even after watch list was announced by WHO. | We thank the reviewer for their useful suggestion. We have indicated in Figure 4 which studies had data including 2017^1,2^ when the Aware Classification was introduced by WHO. Unfortunately, there were only 9 such studies, which prevents us drawing any firm conclusions on the change after introduction of the classification. We have added the following sentence to the “Results, Most frequently use antibiotics”:  “*There were nine studies that included data after 2017, the year when the WHO AWaRe classification was introduced. Quinolones, on the WHO’s Watch List, were still among the top three antibiotics used in Asia, Southern Europe in North America from 2017 onwards*.” (p. 17)  We have also added an introductory sentence to this section with the total number of studies to provide context:  “*There were 59 studies reporting on the most frequently used antibiotics or antibiotic classes*.” (p. 16)  We have also updated the caption for Figure 4 to:  **“Figure 4: Classes of three most frequently used antibiotics reported by studies**.  J01C is penicillins; J01D is cephalosporins; J01M is quinolones; J01X is other classes (includes only methenamine and nitrofurantoin); J01A is tetracyclines; J01F is macrolides. *Classes J01M, J01A and J01F are on the World Health Organization’s (WHO) AWaRe ‘Watch List’ and should be targeted for reduced use due to high resistance potential.*  **Studies including data from 2017 or later. The WHO AWaRe list was first published in 2017*.” (p. 17)  References:   1. Hsia Y, et al. Use of the WHO Access, Watch, and Reserve classification to define patterns of hospital antibiotic use (AWaRe): an analysis of paediatric survey data from 56 coutnries. The Lancet Global Health. 2019; 7(7):e861. 2. WHO. AWaRe. https://aware.essentialmeds.org/groups |
| 5 | Would be interesting to know if appropriate means only the drug or the duration and dosage was also considered. Most of the times the drug appropriateness will be met but the other criteria of stewardship will not be. | We agree that ‘appropriateness’ is measured in different ways and measures different dimensions of antibiotic use. For the appropriateness meta-analysis, we combined studies that used the McGeer Criteria to examine appropriateness. We have added the following text in “Results, Appropriateness of antibiotic therapy” to make clearer what these criteria measure:  “Nine studies, conducted between 2010 and 2018, assessed the appropriateness of antibiotic treatment against the McGeer criteria, *which assess whether antibiotic treatment was warranted based on the presence of infection symptoms*.”  In the Discussion on page 21, first paragraph, we discuss the McGeer criteria and other appropriateness criteria used in the studies. |

**Reviewer 2**

|  | **Comment** | **Response** |
| --- | --- | --- |
| 6 | I must congratulate the authors for a very detailed analysis of an important topic in a neglected patient care setting i.e long term healthcare facilities. The statistical analysis tools are detailed and help bring clarity to the conclusions made. | We thank the reviewer for their comment. |
